# High-Entropy Perovskite Oxide: A New Opportunity for Developing Highly Active and Durable Air Electrode for Reversible Protonic Ceramic Electrochemical Cells

**DOI:** 10.1007/s40820-022-00967-6

**Published:** 2022-11-09

**Authors:** Zuoqing Liu, Zhengjie Tang, Yufei Song, Guangming Yang, Wanru Qian, Meiting Yang, Yinlong Zhu, Ran Ran, Wei Wang, Wei Zhou, Zongping Shao

**Affiliations:** 1 State Key Laboratory of Materials-Oriented Chemical Engineering, College of Chemical Engineering, Nanjing Tech University, Nanjing, 211816 People’s Republic of China; 2 Department of Mechanical and Aerospace Engineering, The Hong Kong University of Science and Technology, Clear Water Bay, Hong Kong, 999077 People’s Republic of China; 3 Institute for Frontier Science, Nanjing University of Aeronautics and Astronautics, Nanjing, 210016 People’s Republic of China; 4 WA School of Mines: Minerals, Energy and Chemical Engineering (WASM-MECE), Curtin University, Perth, WA 6845 Australia

**Keywords:** Reversible proton ceramic electrochemical cells, High-entropy oxide, Air electrode, Oxygen reduction reaction, Oxygen evolution reaction

## Abstract

Reversible proton ceramic electrochemical cell (R-PCEC) is regarded as the most promising energy conversion device, which can realize efficient mutual conversion of electrical and chemical energy and to solve the problem of large-scale energy storage. However, the development of robust electrodes with high catalytic activity is the main bottleneck for the commercialization of R-PCECs. Here, a novel type of high-entropy perovskite oxide consisting of six equimolar metals in the A-site, Pr_1/6_La_1/6_Nd_1/6_Ba_1/6_Sr_1/6_Ca_1/6_CoO_3−*δ*_ (PLNBSCC), is reported as a high-performance bifunctional air electrode for R-PCEC. By harnessing the unique functionalities of multiple elements, high-entropy perovskite oxide can be anticipated to accelerate reaction rates in both fuel cell and electrolysis modes. Especially, an R-PCEC utilizing the PLNBSCC air electrode achieves exceptional electrochemical performances, demonstrating a peak power density of 1.21 W cm^−2^ for the fuel cell, while simultaneously obtaining an astonishing current density of − 1.95 A cm^−2^ at an electrolysis voltage of 1.3 V and a temperature of 600 °C. The significantly enhanced electrochemical performance and durability of the PLNBSCC air electrode is attributed mainly to the high electrons/ions conductivity, fast hydration reactivity and high configurational entropy. This research explores to a new avenue to develop optimally active and stable air electrodes for R-PCECs.

**Supplementary Information:**

The online version contains supplementary material available at 10.1007/s40820-022-00967-6.

## Highlights

- Synthesis of high-entropy perovskite oxide for air electrode in reversible proton ceramic electrochemical cells.
- Triple-conducting high-entropy air electrodes exhibit excellent structural stability and oxygen catalytic activity.
- The peak power density and current density of the cell with high-entropy air electrode in the fuel cell and electrolysis modes are 1.21 W cm^−2^ and − 1.95 A cm^−2^ at 600 °C, respectively.

**Supplementary Information:**

The online version contains supplementary material available at 10.1007/s40820-022-00967-6.

## Graphical abstract

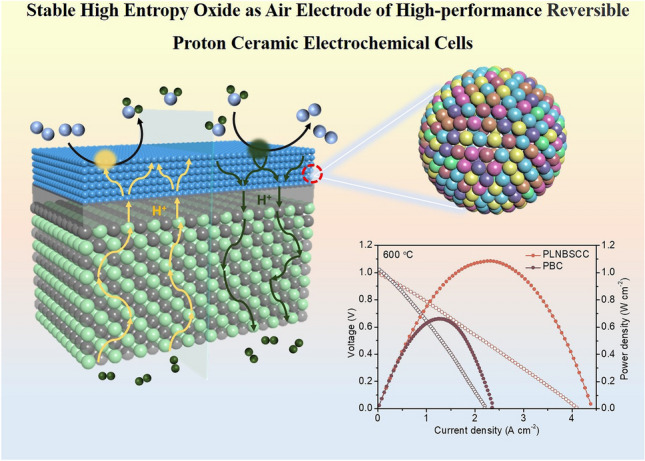

## Introduction

With the continuous exploitation and use of fossil fuels, the problems of energy crisis and environmental degradation are becoming more serious. Renewable energy sources (such as solar, wind and tidal energy) are gradually replacing traditional thermal power supply for electricity generation while alleviating environmental pollution, but these renewable energy faces the mismatch of the supply and demand structure and the difficulty of regional energy dispatch. Consequently, the development of clean, efficient and portable energy conversion devices is highly desirable but currently remains a technological challenge [1]. Compared with traditional solid oxide cells (SOCs) which rely mainly on oxygen-ion conduction, reversible proton ceramic electrochemical cells (R-PCECs) relying on proton conduction have attracted extensive attention due to their low energy consumption and efficient energy conversion [2, 3]. Because proton conduction requires a lower energy barrier, R-PCECs could facilitate the shift in operating temperature from high to intermediate temperatures (500–700 °C), giving rise to improve the durability and mutual matching of various devices of the cells and reduce the cost of raw materials [4–6]. However, one of the technical difficulties for R-PCECs is the insufficient electrocatalytic activity of oxygen reduction reaction (ORR) and oxygen evolution reaction (OER) from air electrodes. Therefore, it is of special significance to develop high-performance bifunctional air electrodes with high electrochemical activity and stability for R-PCECs [7].

Although many mixed ionic (O^2−^) and electronic (*e*^−^) conducting (MIEC) materials, such as SrCo_0.9_Nb_0.1_O_3−*δ*_ (SCN) [8, 9], Ba_0.5_Sr_0.5_Co_0.8_Fe_0.2_O_3−*δ*_ (BSCF) [7, 10] and La_0.4_Sr_0.6_Co_0.2_Fe_0.8_O_3−*δ*_ (LSCF) [11, 12], can be candidates for SOC air electrodes, they are demonstrated to be unsuitable as air electrodes for R-PCECs due to weak proton conductivity and poor tolerance to high concentration of steam [13, 14]. Triple-conducting (*e*^−^/O^2−^/H^+^) oxides (TCO) are the main focus of recent developments in air electrodes for R-PCECs that allow fast transport of electrons, oxygen ions and protons [15, 16]. TCO as air electrodes can extend the active sites from the three-phase boundary (TPB) of the MIEC electrodes to the entire surface, showing great potential for the application of R-PCECs. For example, BaCo_0.4_Fe_0.4_Zr_0.1_Y_0.1_O_3−*δ*_ (BCFZY) was reported as a promising TCO air electrode material with excellent electrochemical performance and durability [17, 18]. PrNi_0.5_Co_0.5_O_3−*δ*_ (PNC) possesses good hydration ability and exhibits commendable electrochemical performance for PCECs in electrolysis mode [19]. Besides the single-phase TCO, nanocomposites prepared by physical composite, impregnation and self-assembly were also designed as promising TCO air electrodes for R-PCECs [20, 21]. Nevertheless, these TCO suffer from the problems of high thermal expansion coefficient, insufficient electrical conductivity and low bifunctionality for both ORR and OER, hence still requiring more advanced air electrodes for R-PCECs [3, 22].

Recently, a new family of metal oxide, high-entropy oxides (HEOs), is attracting growing interest, which offers broad possibilities for adjusting the material functionalities, including electrocatalysis, ionic storage and superconductor [23–25]. Owing to the perovskite crystal structure, the cocktail effect, as well as the synergistic interaction of different ions, high-entropy perovskite oxides (HEPOs) can display some unique structures and catalytic properties and have been successfully used in the application of air electrodes for SOCs [26, 27]. For instance, high- or medium-entropy perovskite oxides La_0.7_Sr_0.3_(Co_0.2_Cr_0.2_Fe_0.2_Mn_0.2_Ni_0.2_)O_3−*δ*_ and Sr(Fe_α_Ti_β_Co_γ_Mn_ζ_)O_3−*δ*_ have been employed as favorable oxygen electrodes [28, 29]. Unfortunately, the increase of B-site configuration entropy can effectively improve the structural stability of electrode materials, but inevitably sacrifices the electrocatalytic activity of SOC cathodes. To address this issue, some A-site configuration HEPOs, such (La_0.2_Pr_0.2_Nd_0.2_Sm_0.2_Gd_0.2_)_2_CuO_4_ and La_0.2_Pr_0.2_Nd_0.2_Sm_0.2_Sr_0.2_MnO_3−*δ*_, were also designed and reported to significantly improve the oxygen-ion conductivity and overall electrochemical activity of air electrodes [30, 31]. Compared with common perovskites, the introduction of more metal cations into the specific lattice of perovskites provides abundant possibilities for better design and tailoring of catalysts. This facilitates the excellent activity of the electrode catalyst in various catalytic reaction processes (ORR and OER). Meanwhile, the uniform distribution of various cations further enhances the entropy-dominated stabilization effect in the perovskite oxide, which will improve the tolerance of the catalyst under complex operating conditions. Therefore, under this premises, combining the concept of high entropy with perovskite oxides to form HEPOs opens new opportunities for designing high-performance bifunctional air electrodes for R-PCECs.

Inspired by aforementioned considerations, here we demonstrate for the first time a high-entropy perovskite oxide, Pr_1/6_La_1/6_Nd_1/6_Ba_1/6_Sr_1/6_Ca_1/6_CoO_3−*δ*_ (PLNBSCC) derived from the PrBaCo_2_O_5−+*δ*_ (PBC) prototype, as a highly ORR and OER active and stable air electrodes for R-PCECs. Due to the unique properties of rare earth and alkaline earth oxides with good ionic and electronic conductivity and promoting hydration reaction, under the synergistic strengthening of the abundant trivalent rare earth elements and divalent alkaline earth metal elements at the A-site, the triple conductivity and hydration ability of PLNBSCC are significantly improved. When the PLNBSCC air electrode was applied to a single cell supported by a Ni-BaZr_0.1_Ce_0.7_Y_0.1_Yb_0.1_O_3−*δ*_ (BZCYYb) fuel electrode, promising stability and exceptional performances have been demonstrated in both modes at 600 °C, with peak power density of 1.21 W cm^−2^ in fuel cell operation and current density of − 1.95 A cm^−2^ under the electrolysis operation (1.3 V), while retaining robust operation at high steam concentrations and thermal cycling tolerance. Our findings provide a new framework for developing advanced R-PCEC electrodes via designing high-entropy structures.

## Experimental Section

### Materials Synthesis and Cell Fabrication

The high-entropy perovskite oxide PLNBSCC was synthesized via the sol–gel method. Pr(NO_3_)_3_·6H_2_O, La(NO_3_)_3_·6H_2_O, Nd(NO_3_)_3_·6H_2_O, Ba(NO_3_)_2_, Sr(NO_3_)_2_ Ca(NO_3_)_2_·6H_2_O and Co(NO_3_)_2_·6H_2_O were initially dissolved in deionized water. Then, ethylenediaminetetraacetic acid (EDTA) and citric acid (CA) as complexing agents were employed with a 2:1:1 mol ratio for citric acid/total metal ions/EDTA. The pH value of the solution adjusted to ~ 7 by adding NH_3_ aqueous solution. The solution was stirred and heated at 90 °C until it became a transparent gel. The gel-like intermediate product was heated at 200 °C for 10 h to obtain the precursor, which was placed in a chamber furnace and calcined at 1000 °C for 5 h to obtain the electrode powder. Other electrodes and electrolyte powders were prepared by the same method. Anode powders consisting of BZCYYb, NiO and starch with a 3.5:6.5:1 mass ratio were prepared by ball milling (pulverisette 6, FRITSCH) and dried. Then, the half-cells with the configuration of Ni-BZCYYb/BZCYYb anode supported were fabricated by co-pressing and high-temperature sintering at 1450 °C for 5 h. Furthermore, Gd_0.2_Ce_0.8_O_1.9_ (GDC) and BZCYYb electrolyte disks were prepared by dry pressing and sintering at 1350 and 1400 °C for 10 h, respectively. The spin-coating method to fabricate half-cells is described as follows. The same anode powder (0.35 g) as described above was dry-pressed (10 MPa) into a disk shape and calcined at 1000 °C in still air for 2 h to obtain a pretreated anode support layer. Electrolyte slurry was prepared by ball milling of BZCYYb, ethyl cellulose, ethanol and ethylene glycol according to the ratio of 1 g:0.1 g:10 mL:2 mL for 1 h. Subsequently, the anode support layer was fixed on the spin coater, the electrolyte slurry was covered with the anode support layer, and spin coating was performed at 8000 rpm for 30 s, which was repeated three times, and the obtained half-cell was sintered at 1450 °C for 5 h.

The electrode powder, isopropanol, ethylene glycol and glycerol were added to the ball mill tank according to the ratio (1 g:10 mL:2 mL:1 mL) and ball milled at 400 rpm for 30 min to obtain cathode slurry. Subsequently, the slurry was sprayed on the electrolyte side of the half-cell and both sides of the electrolyte disk, respectively, and sintered at 1000 °C for 2 h to obtain single cells (Ni-BZCYYb/BZCYYb/electrode) and symmetrical cells (electrode/BZCYYb/electrode). It was important to note that the effective area of the air electrode was limited to 0.25 cm^−2^ in a single cell. A sliver pastes and mesh-like sliver were also painted onto the surface of electrode for electrochemical impedance spectroscopy (EIS) test and area specific resistance (ASR) stability test, respectively. To evaluate the oxygen-ion and electronic conductivities of PLNBSCC and PBC electrodes, PLNBSCC and PBC powders were pressed into pellets and disks, respectively, and calcined at 1190 and 1200 °C for 10 h, respectively. Dense rods and pellets (polished to a thickness of 0.6 mm) were used for electrical conductivity testing and oxygen permeability testing, respectively.

### Electrochemical Testing

In PCFC mode, the air electrode side was exposed to flow air (100 mL min^−1^), and the anode side was fed with dry hydrogen gas at flow rate of 80 mL min^−1^. In PCEC mode, steam was mixed into the air at different flow rates by a high-pressure constant flow pump, and the total gas flow rate for the air electrode was 100 mL min^−1^. In addition, the rate of hydrogen production by electrolysis was tested using hydrogen as reduction anode, followed by argon (60 mL min^−1^) as the carrier gas. Meanwhile, hydrogen production was estimated using gas chromatograph (GC-9860-5CNJ) with in situ thermal conductivity detector (TCD). The *I*–*V* and power density curves of cells were captured by fuel cell test workstation (Keithley 2420) based on a four-probe configuration. The conductivities of the different electrodes were measured under dry or humid air conditions at a flow rate of 100 mL min^−1^ by a four-probe direct current method using a Keithley 2420. EIS measurements of single cells and symmetric cells were performed using an electrochemical workstation (Solartron 1287 + 1260A). Among them, the EIS of the fuel cell and electrolysis modes were performed at the open circuit voltage (OCV) and the nominal voltage of 1.3 V, respectively.

### Basic Characterizations

Ambient X-ray diffraction (XRD) of powders was acquired by Bruker D8 Advance, and in situ high-temperature XRD were performed for sample structure characterization by high-temperature accessories (Rigaku D/max 2500 V). Surface and cross-sectional topography of powders and cells were obtained by scanning electron microscopy (SEM) (JEOL-S4800). High-resolution transmission electron microscopy (HRTEM) images, high-angle annular dark-field (HAADF) scanning transmission electron microscopy (STEM) images along with the corresponding energy-dispersive X-ray (EDX) elemental mapping were carried out with a FEI talos F200× G2 and super-x instrument. The surface element states were probed using X-ray photoelectron spectroscopy (XPS) at room temperature (Thermo ESCALAB 250). Thermogravimetric analysis (TGA, Model STA 449 F3, NETZSCH) was used to detect changes in the mass of the sample over time and temperature. The in situ infrared (IR) spectra of the perovskite oxides were taken on an IR spectrometer (IS50 FTIR) and an in situ diffuse reflectance test cell containing an MCT detector in wet air atmosphere with a temperature rate of 5 °C min^−1^.

## Results and Discussion

### Analysis of Composition and Structure

**Fig. 1:**
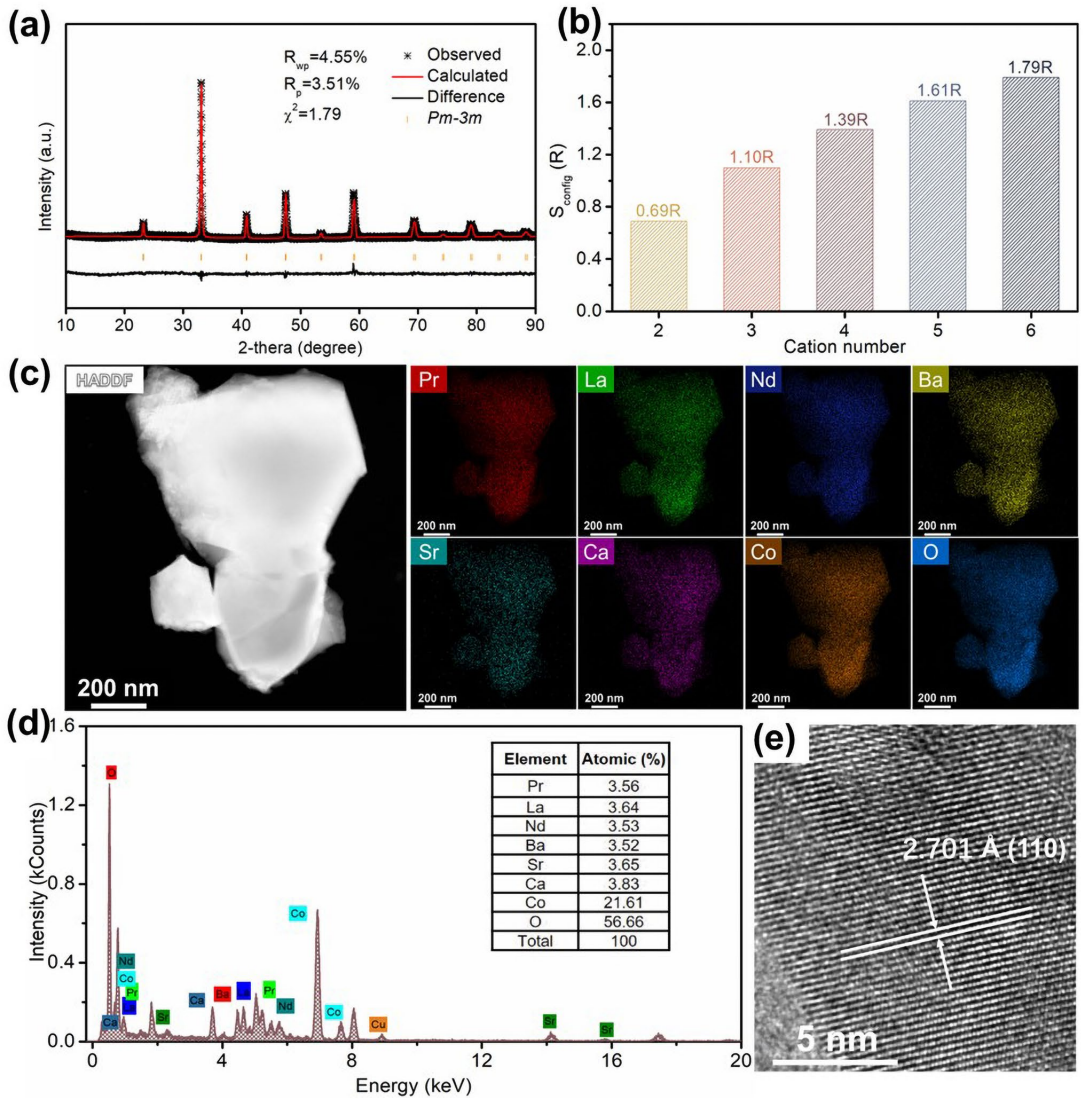
**a** Refined XRD profile of PLNBSCC powder. **b** Dependence of the maximum configurational entropy of metal oxide systems on the number of cations contained. **c** TEM image and EDX elemental mapping of PLNBSCC powder. **d** EDX spectra of PLNBSCC particle in Figure **c**. **e** HRTEM image of PLNBSCC

The PLNBSCC and PBC perovskite oxides were synthesized by a facile sol–gel method and the microscopic morphologies were initially observed by SEM (Fig. S1). The PLNBSCC oxide was confirmed its crystallization as a single-phase cubic perovskite structure by XRD measurements (Fig. S2). In addition, XRD refinement data further revealed that PLNBSCC is a space group of *Pm-3m* with lattice parameters of *a* = *b* = *c* = 3.8301(3) Å (Fig. 1a, *R*_p_ = 3.51%, *R*_wp_ = 4.55%, *χ*^2^ = 1.79). Meanwhile, the parent oxide PBC was shown a typical layered perovskite structure, space group *P4/mmm*, with *a* = *b* = 3.9103(6) Å, and *c* = 7.6557(6) Å after Rietveld refinement (Fig. S3, Table S1) [32, 33]. Here, the transformation of the crystal structure from layered perovskite to cubic perovskite is realized by increasing the number of A-site cation species of PBC, which is based on the entropy stabilization effect generated by the increase in the configurational entropy of the perovskite oxide system. The configurational entropy of the oxide system can be calculated by Eq. (1) [34, 35].

1 $$S = - R\left[ {\left( {\mathop \sum \limits_{a = 1}^{A} x_{a} \ln x_{a} + \mathop \sum \limits_{b = 1}^{B} x_{b} \ln x_{b} } \right)_{{\text{cation - site}}} + \left( {\mathop \sum \limits_{j = 1}^{M} x_{j} \ln x_{j} } \right)_{{\text{anion - site}}} } \right]$$

where *A*, *B* and *M* represent the number of element species at the A-site cation, B-site cation and anion sites, respectively, *x*_*a*_, *x*_*b*_ and *x*_*j*_ represent the mole fraction of the corresponding elements, and *R* is the gas constant [25]. Therefore, when the atomic fractions of all elements are equal, the doping number versus configurational entropy of A-site cations is shown in Fig. 1b (the influence of possible oxygen vacancies and electron holes is not taken into consideration). Obviously, the A-site configuration entropy of PLNBSCC is 1.79R, while the configurational entropy of PBC oxide as a simple double perovskite is 0, since the Pr and Ba positions can be clearly divided. Therefore, PLNBSCC and PBC are regarded as high-entropy and low-entropy perovskite oxides, respectively [36]. It is noteworthy that the increase of configuration entropy enables the stable formation of cubic structure of PLNBSCC oxide material at lower temperatures (Fig. S4). PBC oxide exhibited characteristic peaks of BaCoO_3_ and PrCoO_3_ at 700 and 800 °C, and poor crystallinity compared to PLNBSCC at 900 and 1000 °C, which indicates that the enhancement of configurational entropy can drive the structural stability [35]. Furthermore, to probe the distribution of elements in PLNBSCC, the homogeneity of Pr, La, Nd, Ba, Sr, Ca, Co and O elements was revealed by HAADF-STEM image and EDX elemental mapping (Fig. 1c). Moreover, the EDX spectrum shown in Fig. 1d indicates that the atomic ratio of each element is close to the theoretical stoichiometric ratio. The cubic configuration of the high-entropy perovskite oxide PLNBSCC was then investigated by HRTEM. The lattice spacing shown in Fig. 1e is 2.701 Å, which corresponds to the (110) lattice plane of PLNBSCC oxide.

### Electrochemical Performance of Symmetrical Cells

**Fig. 2:**
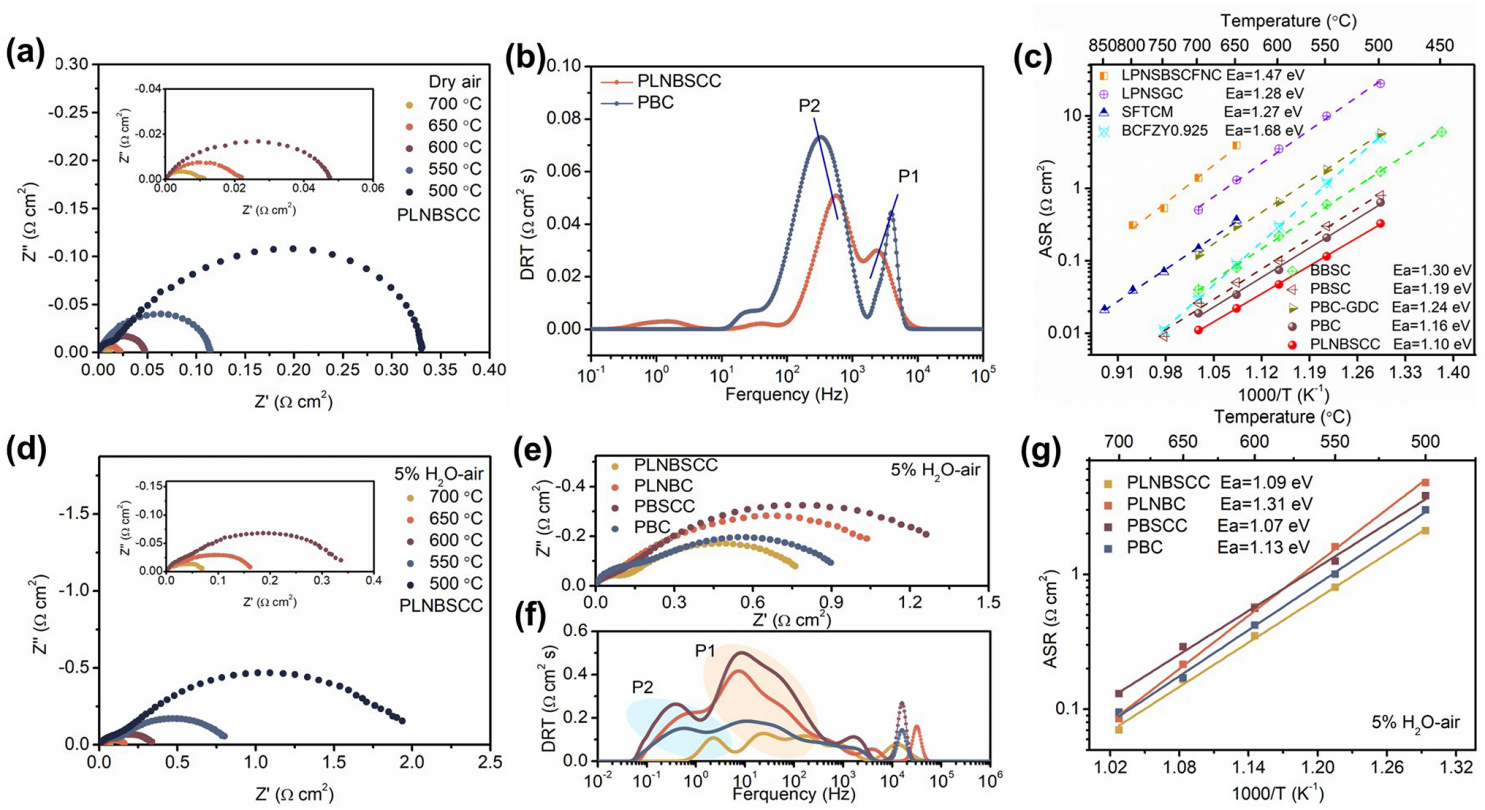
**a** EIS curves of GDC based-supported symmetrical cell with PLNBSCC measured at 500, 550, 600, 650 and 700 °C. **b** DRT analysis of EIS spectra of PLNBSCC and PBC electrodes at 600 °C. **c** Arrhenius plots of the *R*_p_ of the PLNBSCC, PBC, LPNSBSCFNC, LPNSGC, SFTNM, BCFZY0.925, BBSC, PBSC and PBC-GDC electrodes. **d** EIS curves of BZCYYb-based symmetric cell with PLNBSCC electrode tested at 700–500 °C in 5% H_2_O–air. **e** EIS comparison of PLNBSCC, PLNBC, PBSCC and PBC electrodes at 550 °C with 5% H_2_O–air, and **f** corresponding DRT plots. **g** Activation energy plots of symmetrical cells with PLNBSCC, PLNBC, PBSCC and PBC electrodes under 5% H_2_O–air at 500, 550, 600, 650 and 700 °C

The remarkable kinetic rate of oxygen reduction reaction at the air electrode is the guarantee for R-PCECs to obtain high electrochemical performance in fuel cell mode. EIS of symmetric cells supported by Gd_0.2_Ce_0.8_O_1.9_ (GDC) electrolyte were firstly tested, which was stripped the effect of the proton conduction of the electrode on the ORR activity [4, 37]. Figure 2a shows EIS curves of symmetric cells with PLNBSCC electrode tested at 700, 650, 600, 550 and 500 °C, with polarization resistances (*R*_p_) of 0.01, 0.02, 0.05, 0.12 and 0.33 Ω cm^2^, respectively. However, the *R*_p_ of the PBC air electrode were significantly higher than that of the PLNBSCC under the same conditions (Fig. S5). The XRD patterns indicated that no obvious phase reaction was found after calcination of the air electrode and the electrolyte in equal mass, ensuring that the experimental results are valid (Fig. S6). Moreover, it is well known that there are many factors affecting the ORR kinetics on the surface of air electrode and the reaction process is intricate. Distribution of relaxation time (DRT) is an efficient tool to deconvolute EIS spectrum to better explore the rate-determining step of the electrochemical reaction at the air electrode [38–40]. Figure 2b shows the DRT fitting curves of the EIS data obtained for PLNBSCC and PBC electrodes in air at 600 °C. DRT plots can generally be divided into high-frequency (HF) region, intermediate-frequency (IF) region and low-frequency (LF) region according to the frequency range and represent the electron and ion migration of electrodes, surface ion exchange and gas diffusion, respectively. As shown in Fig. 2b, the areas of HF peak P1 and IF peak P2 of PLNBSCC air electrode are lower than those of PBC electrode, indicating that the high-entropy PLNBSCC oxide has faster charge transfer and the bulk diffusion of oxygen is also greatly improved [41]. Moreover, at the same temperature, the *R*_p_ values of PLNBSCC are substantially lower than the reported air electrode materials, including La_0.2_Pr_0.2_Nd_0.2_Sm_0.2_Ba_0.1_Sr_0.1_Co_0.2_Fe_0.6_Ni_0.1_Cu_0.1_O_3−*δ*_ (LPNSBSCFNC), (La_0.2_Pr_0.2_Nd_0.2_Sm_0.2_Gd_0.2_)_2_CuO_4_ (LPNSGC), SrFe_0.25_Ti_0.25_Co_0.25_Mn_0.25_O_3−*δ*_ (SFTCM), Ba(Co_0.4_Fe_0.4_Zr_0.1_Y_0.1_)_0.975_O_3−*δ*_ (BCFZY0.925), Ba_2_Bi_0.1_Sc_0.2_Co_1.7_O_6-δ_ (BBSC), PrBa_0.5_Sr_0.5_Co_2_O_5+*δ*_ (PBSC) and PBC-GDC electrodes (Fig. 2c) [4, 10, 30, 31, 42–45]. Meanwhile, the lowest activation energy exhibited by the PLNBSCC air electrode also confirms that the ORR activity is less temperature-dependent, suggesting that the R-PCECs with PLNBSCC electrode has the potential to be applied at lower temperatures.

The hydration reaction of the air electrode is an important reaction process that determines the proton introduction ability and the mass transfer on the electrode surface [18, 46]. The EIS of BZCYYb electrolyte-supported symmetric cells with different air electrodes tested in argon atmosphere with 5 vol% water pressure (H_2_O-Ar) was used to investigate their hydration reactivity. EIS curves of PLNBSCC and PBC electrodes are shown in Fig. S7a–b, the *R*_p_ of PLNBSCC and PBC are 4.7 and 7.5 Ω cm^2^ at 600 °C, respectively.

The lower *R*_p_ and activation energy indicates that high-entropy PLNBSCC oxide has better hydration capacity compared with PBC oxide (Fig. S8). In addition, the EIS of PLNBSCC and PBC was also measured under dry air and shown in Fig. S9a. Meanwhile, (Pr_1/3_La_1/3_Nd_1/3_)_0.5_Ba_0.5_CoO_3−*δ*_ (PLNBC) and Pr_0.5_(Ba_1/3_Sr_1/3_Ca_1/3_)_0.5_CoO_3−*δ*_ (PBSCC) were also successfully synthesized (Figs. S10 and S11), and the respective EIS was obtained under the same conditions (Fig. S9c, d). In contrast, the lowest *R*_p_ of the PLNBSCC electrode were confirmed excellent ORR activity on proton conducting symmetric cell (Fig. S12).

To investigate the electrochemical activity of the air electrode catalysts under practical operating conditions, the EIS curves of PLNBSCC, PLNBC, PBSCC and PBC air electrodes at 5% H_2_O–air were obtained at 700–500 °C (Figs. 2d and S13a–c). As shown in Fig. 2d, the *R*_p_ of the high-entropy PLNBSCC air electrode are 0.07, 0.16, 0.35, 0.81 and 2.09 Ω cm^2^ at 700, 650, 600, 550, and 500 °C, respectively. Compared with dry air conditions, the introduction of water vapor greatly reduces *R*_p_, because the hydration reaction accelerates the surface ion exchange and bulk proton conductivity [47]. Nevertheless, the PLNBSCC air electrode still has the lowest *R*_p_ values compared to the PLNBC, PBSCC and PBC air electrodes, and their EIS curves are also compared at 550 °C in Fig. 2e. The deconvolution peaks fitted by DRT can be clearly observed that the areas of the intermediate-frequency peak P1 and the low-frequency peak P2 of the PLNBSCC electrode are lower than those of the other electrodes (Fig. 2f). This indicates that the excellent ORR activity of the high-entropy PLNBSCC electrode is due to the fast reaction kinetics, which are closely related to the fast electron and ion conduction and the hydration reaction. The Arrhenius plots in Fig. 2g indicate that the high-entropy PLNBSCC electrode has a lower activation energy, which implies its feasibility for low-temperature applications (Table S2) [15, 48]. In addition, in order to reveal the optimization effect on the overall structure–activity of PBC with the gradual introduction of each element, (Pr_1/3_Ba_1/3_Ca_1/3_)_2_Co_2_O_5+*δ*_ (PBCC), Pr_1/4_La_1/4_Ba_1/4_Ca_1/4_CoO_3−*δ*_ (PLBCC) and Pr_1/5_La_1/5_Ba_1/5_Sr_1/5_Ca_1/5_CoO_3−*δ*_ (PLBSCC) electrodes were synthesized, and the ASRs of their symmetric cells were obtained under 5% H_2_O–air condition. The results show that with the sequential addition of Ca, La, Sr and Nd, the configurational entropy of the electrode gradually increases, which is accompanied by the stabilization of the structure and the enhancement of the activity (Fig. S14 and Table S3). Furthermore, the stability test of the symmetric cell with PLNBSCC as the air electrode at 600 °C with 5% H_2_O–air has a lower decay rate than the layered perovskite PBC electrode (Fig. S15). At the same time, the high-temperature in situ XRD patterns and room-temperature XRD patterns shown in Figs. S16 and S17 indicate good structural stability and phase compatibility with electrolyte, respectively [49].

### Conductivity and Hydration Reaction

**Fig. 3:**
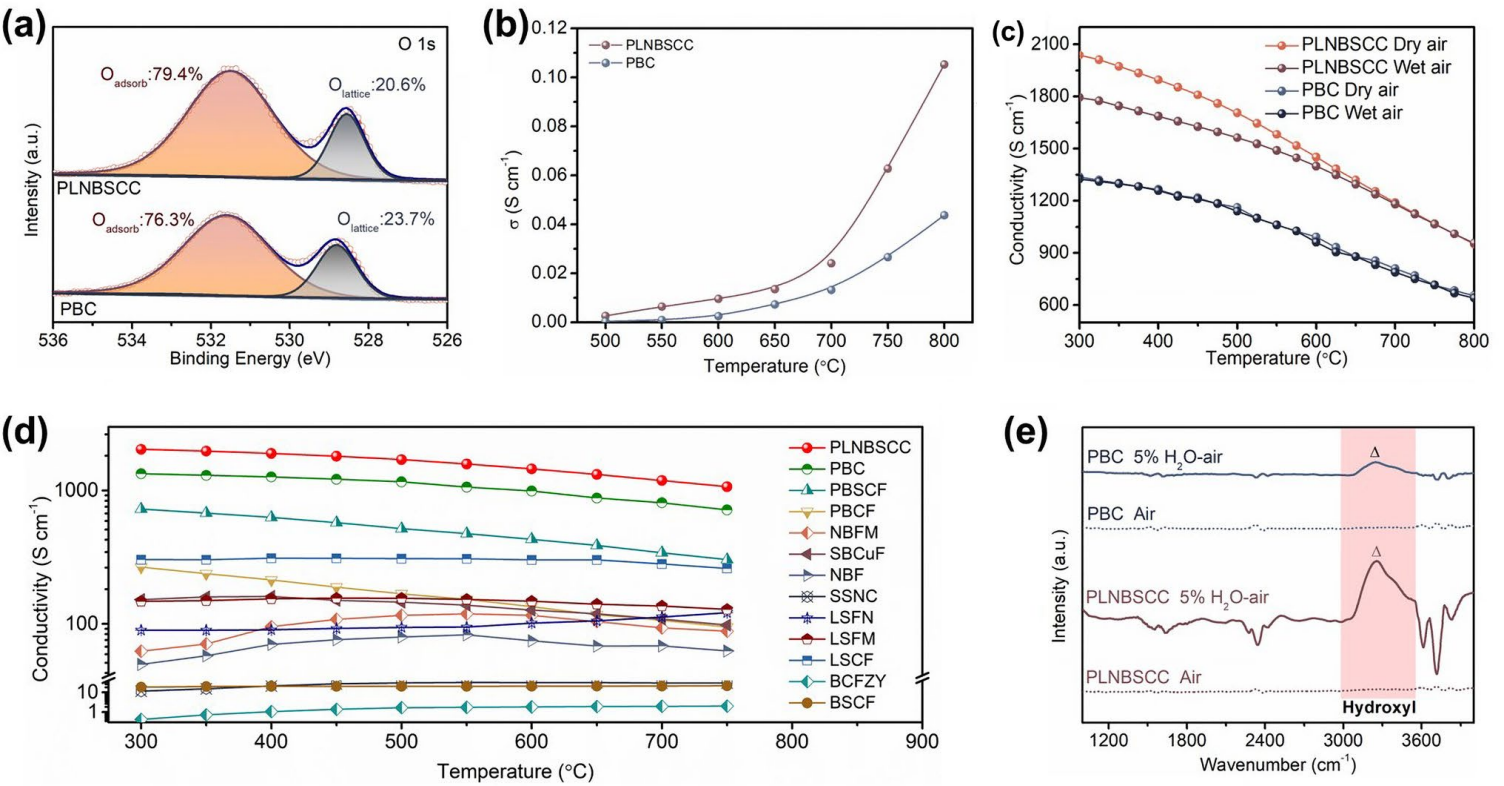
**a** XPS spectra of O 1*s* of PLNBSCC and PBC oxides. **b** O^2−^ conductivities of PLNBSCC and PBC oxides in the temperature range of 500–800 °C. **c** Electrical conductivity of PLNBSCC and PBC oxides measured under dry and wet air (3% H_2_O) from 300 to 800 °C. **d** Electrical conductivity of PLNBSCC compared to most typical air electrodes at 300–750 °C. **e** IR spectra of PLNBSCC and PBC oxides after treatment at 400 °C for 2 h in dry air and 5% H_2_O–air

The underlying reasons for the significantly enhanced ORR activity of the high-entropy air electrode PLNBSCC compared with PBC were further explored. Oxygen vacancies (O_v_) content as an index to measure the activity and rate of electrochemical reaction is because the surface electrochemical reaction and bulk ion transport of air electrode depend on O_v_. The oxygen non-stoichiometries of PLNBSCC and PBC oxides were determined by iodometric titration method to be 0.252 and 0.323, respectively [43]. Since the PLNBSCC is a single perovskite structure, the PLNBSCC oxide have more oxygen vacancy content under the same mass condition. Furthermore, probing the state of oxygen on the surface of the air electrodes helps to reveal the evolution of its electrochemical reaction [50, 51]. Figure 3a shows XPS spectra of the O 1*s* of PLNBSCC and PBC samples at room temperature. The surface adsorbed oxygen (O_adsorb_) and lattice oxygen (O_lattice_) accounted for 79.4% and 20.6% of the PLNBSCC sample, respectively. The deconvolution peak area ratio of O_adsorb_ to O_lattice_ of PLNBSCC (3.85) is much higher than that of PBC (3.22) [52]. Moreover, the loss of oxygen at high temperature was also characterized by TGA (Fig. S18). However, PLNBSCC oxide exhibited slightly higher mass loss than PBC. Overall, the high-entropy PLNBSCC air electrode has abundant oxygen vacancies to ensure the rapid progress of the electrochemical reaction. Therefore, it is reasonable to infer that the enhanced electrochemical activity of the high-entropy PLNBSCC oxide is partly due to the modification of its surface oxygen species and abundant oxygen vacancy content [53, 54].

In addition, the oxygen-ion (O^2−^) conductivities of PLNBSCC and PBC were also obtained through an oxygen permeable membrane with a thickness of ~ 0.7 mm at 500–800 °C. The oxygen permeability of PLNBSCC in the tested temperature range is higher than that of layered perovskite oxide PBC (Fig. S19), indicating its higher O^2−^ conductivity (Fig. 3b). For example, at 750 °C, the O^2−^ conductivity is 0.064 S cm^−1^ for PLNBSCC electrode, while the O^2−^ conductivity is 0.026 S cm^−1^ for PBC electrode, respectively. Compared with the ionic conductivity, the electronic conductivity of perovskite oxides dominates the total conductivity and plays a crucial role in the ORR and OER processes of air electrodes. The conductivities of PLNBSCC and PBC oxides in dry and humid air atmospheres obtained by 4-probe DC conductivity method are revealed in Fig. 3c. In the temperature range of 500–800 °C, the conductivity of high-entropy PLNBSCC oxide increases from 950 to 2038 S cm^−1^ in dry air, while that of PBC increases from 650 to 1330 S cm^−1^. Therefore, the conductivity of PLNBSCC completely outperforms than that of PBC. Furthermore, the conductivity of the PLNBSCC air electrode shows outstanding advantages compared to the best-known advanced air electrodes, e.g., in the temperature range of 750–300 °C, the conductivities of perovskite oxides BCFZY, BSCF, LSCF and PrBa_0.5_Sr_0.5_Co_1.5_Fe_0.5_O_5+δ_ (PBSCF) are 0.4–2, 18–21, 260–302 and 303–726 S cm^−1^, respectively (Fig. 3d) [55–63]. Therefore, such excellent high conductivity of PLNBSCC provides a guarantee for commercialization. Importantly, the conductivity of PLNBSCC oxide in wet air condition is lower than that measured in air below 675 °C. On the contrary, there was no significant change in PBC oxide. This is because in humid atmosphere, the PLNBSCC oxides introduce proton conductivity through hydrogenation reaction, resulting in some electron holes being occupied (Eq. 2) [51, 64].

2 $${\text{H}}_{2} {\text{O}} + 2{\text{O}}_{{\text{O}}}^{ \times } + 2h^{ \cdot } \leftrightarrow 2{\text{OH}}_{{\text{O}}}^{ \cdot } + 1/2{\text{O}}_{2}$$

where the ${\text{O}}_{{\text{O}}}^{ \times }$, *h*^·^ and ${\text{OH}}_{{\text{O}}}^{ \cdot }$ represent lattice oxygen, electron holes and hydroxide formed by the attachment of protons to lattice oxygen, respectively. Thus, the high-entropy oxide PLNBSCC can again be confirmed as a triple conductor with electrons, oxygen ions and protons. In addition, the hydration reaction uses oxygen vacancies as reaction sites to dissociate water and form hydroxides based on lattice oxygen, thus providing a prerequisite for proton conduction. To characterize the hydration ability of PLNBSCC and PBC oxides, the high-temperature in situ IR spectra were measured as shown in Fig. 3e [7, 65]. No obvious hydroxyl peaks were found in PLNBSCC and PBC oxides in dry air at 400 °C (3000–3600 cm^−1^), and then treated in 5% H_2_O–air atmosphere for 2 h, PLNBSCC oxide had more obvious hydroxyl characteristic peak compared with PBC oxide, indicating that PLNBSCC has excellent hydration reactivity [39]. Therefore, the excellent electrochemical activity and reaction kinetic rate of the high-entropy PLNBSCC electrode is attributed to the excellent electrons/ions conductivity and hydration reactivity.

### Performance and Stability of R-PCECs

**Fig. 4:**
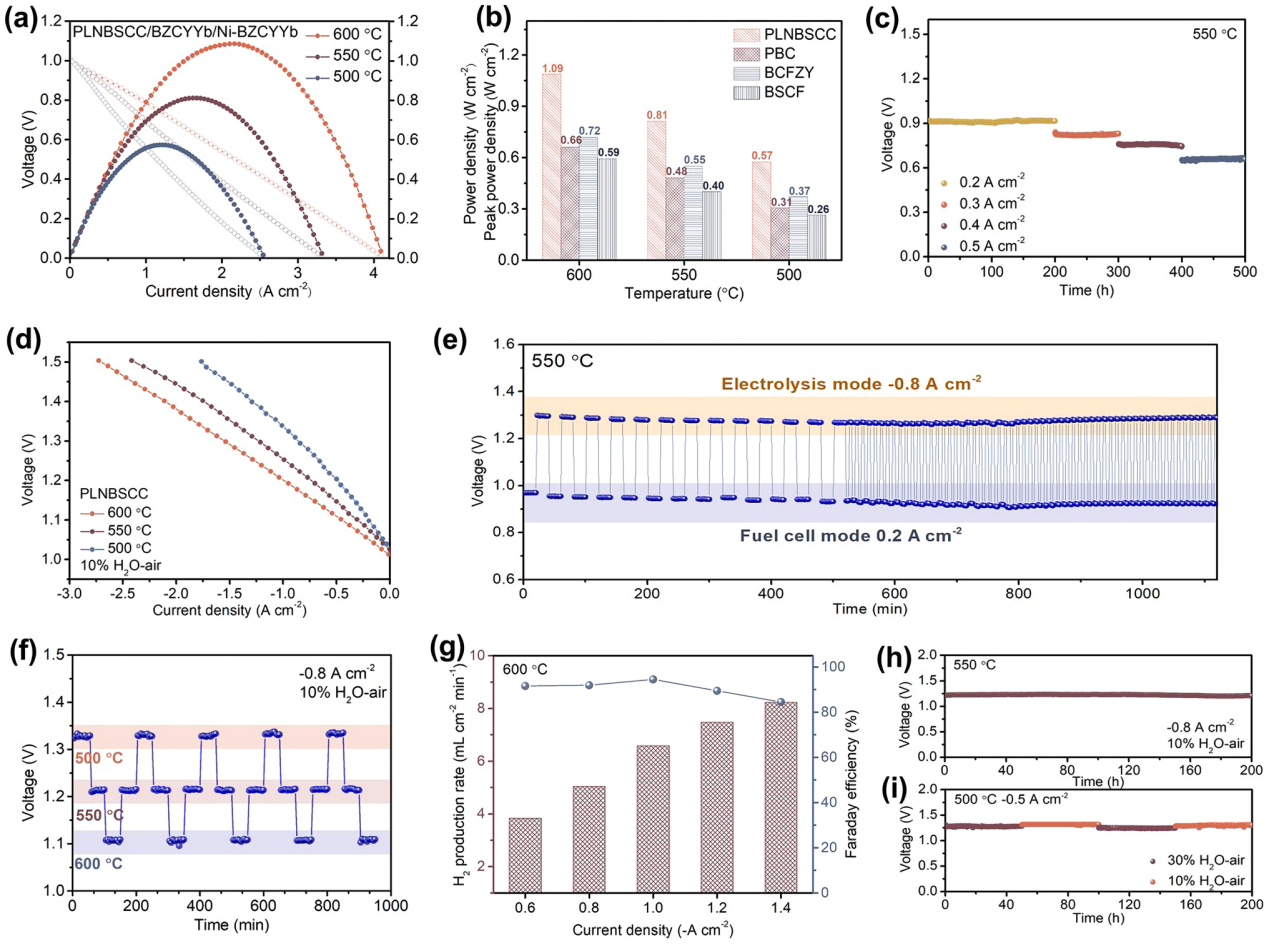
**a** *I*–*V* and power density curves of single cell with high-entropy PLNBSCC as the air electrode at 500, 550 and 600 °C. **b** Peak power densities of PLNBSCC air electrode in fuel cell mode compared to PBC, BCFZY and BSCF air electrodes. **c** Stability of PLNBSCC cell in fuel cell mode at different current densities (0.2, 0.3, 0.4 and 0.5 A cm^−2^) and 550 °C. **d** Typical *I*–*V* curves of single cell with PLNBSCC electrode tested in electrolysis mode at 500–600 °C. **e** The voltages were observed in reversible operation of R-PCECs at 550 °C. **f** Thermal cycling durability test in electrolysis mode at 500–600 °C. **g** Hydrogen production rate and FE as function of current densities at PLNBSCC electrode in electrolysis mode with argon in the fuel electrode and 30% H_2_O–air in the air electrode at 600 °C. **h** Stability test of single cell in electrolysis mode at galvanostatic current density − 0.8 A cm^−2^ and 550 °C. **i** Electrolysis cell durability test under different water pressure conditions (10% and 30% H_2_O–air) at 500 °C

The excellent electrochemical activity and entropy-driven stabilization of the high-entropy PLNBSCC air electrode was further confirmed by R-PCEC. SEM micrograph of the section of an as-prepared single cell with a porous support layer Ni-BZCYYb as fuel electrode, dense BZCYYb electrolyte with a thickness of 19 µm and PLNBSCC air electrode is shown in Fig. S20. Figure 4a demonstrates the typical *I*–*V* and power density curves of a single cell using PLNBSCC air electrode with extraordinary peak power densities (PPDs) of 1.09, 0.81 and 0.57 W cm^−2^ at 600, 550 and 500 °C in fuel cell operation, respectively. Furthermore, the PPDs of the single cell with double perovskite oxide PBC as air electrode were also obtained as shown in Fig. S21a. At the same time, R-PCECs with the well-recognized BCFZY and BSCF air electrodes were also tested under the same conditions (Fig. S21b–c). Figure 4b depicts the comparison of PPDs obtained with PLNBSCC, PBC, BCFZY and BSCF electrodes. The PPDs of 0.66, 0.72 and 0.59 W cm^−2^ were obtained for PBC, BCFZY and BSCF electrodes at 600 °C, and compared with these electrodes, the PPD of PLNBSCC electrode is increased by 65%, 51% and 85%, respectively. The remarkable improvement of the electrochemical performance of the single cell with PLNBSCC air electrode is inseparable from the contribution of excellent ORR activity and high conductivity. Figure 4c exhibits the voltage characteristics of the single cell with PLNBSCC air electrode at different current densities (0.2, 0.3, 0.4 and 0.5 A cm^−2^) for fuel cell operation. Notably, the single cell still maintained stable voltage feedback without significant degradation in the endurance test up to 500 h, this suggests that the increase in entropy drives the stability of the electrode [66]. Furthermore, the porous PLNBSCC electrode provided good gas diffusion and maintained close contact with the electrolyte layer, which ensured stable operation of the single cell (Fig. S22) [42].

The typical *I*–*V* curves of R-PCEC with PLNBSCC air electrode were obtained under 10% H_2_O–air to further evaluate the OER activity and electrolysis performance of the electrode. As shown in Fig. 4d, at 600, 550 and 500 °C, the current densities of electrolysis corresponding to a voltage of 1.3 V were − 1.53, − 1.21 and − 0.84 A cm^−2^, respectively. To its credit, the electrolysis performance achieved by the PLNBSCC electrode is one of the highest measurements of R-PCEC reported so far. As the water pressure increased to 30%, the current density obtained by the cell in electrolysis mode increased to − 1.73 A cm^−2^, indicating the excellent hydration and water adsorption capacity of the PLNBSCC electrode (Fig. S23). Meanwhile, the current densities obtained for the PBC electrode were − 1.09, − 0.80 and − 0.53 A cm^−2^ (1.3 V) at 600, 550 and 500 °C, respectively (Fig. S24a). The *I*–*V* curves of BCFZY and BSCF air electrodes in electrolysis mode were also measured at 500–600 °C (Fig. S24b, c). The current densities of single cell with PLNBSCC electrode were higher than those of the cells with PBC, BCFZY and BSCF as air electrodes (e.g., 40%, 33% and 101% at 600 °C, respectively) (Fig. S24d). However, the stability of the reversible operation of R-PCECs in both electrolysis and fuel cell modes is an important indicator to measure the feasibility of in practical operation [67, 68]. As shown in Fig. 4e, the cell was first run in fuel cell mode for 20 min (0.2 A cm^−2^), and subsequently switched to hydrogen production mode for consistent run time under 10% H_2_O–air (− 0.8 A cm^−2^). After 500 min of continuous operation, a fast dynamic response per cycle of 10 min was performed. The single cell with PLNBSCC air electrode maintained stable operation without obvious decay during the 1120-min cycle test. On the other hand, the single cell with PLNBSCC electrode also maintained a relatively stable performance output under different voltage cycling tests (Fig. S25).

The degradation of the electrocatalysis activity of the air electrode with the prolonged fluctuation of the test temperature is still a drawback faced by most electrodes. As shown in Fig. 4f, during 5 cycles from 500 to 600 °C, the single cell was operated at a consistent current density of − 0.8 A cm^−2^, and the corresponding feedback voltage values remain stable at different temperatures, indicating the good thermal matching and compatibility between the PLNBSCC electrode and the electrolyte (Fig. S26). The excellent electrolytic activity of the high-entropy air electrode PLNBSCC was further demonstrated by testing its hydrogen production rate and Faradaic efficiency (FE) under different current densities at 600 °C and 30% H_2_O–air. The hydrogen production rates were 3.83, 5.04, 6.58, 7.47 and 8.23 mL cm^−2^ min^−1^ at current densities of − 0.6, − 0.8, − 1.0, − 1.2 and − 1.4 A cm^−2^, respectively, and corresponding FE reached more than 80% (Fig. 4g) [3, 19, 69]. Besides, the long-term (200 h) stability test of a single cell under electrolysis operation was performed to verify the durability of the high-entropy PLNBSCC air electrode, as shown in Fig. 4h, and the smooth voltage feedback indicates the excellent electrochemical stability of PLNBSCC electrode. At the same time, it also performed well within 200 h in the tolerance test at different water pressures (30% and 10%) (Fig. 4i). In addition, the high-entropy PLNBSCC air electrode was treated at 550 °C for 24 h in a 30% H_2_O–air atmosphere and still maintained good structural stability, and no extra impurity peaks were found (Fig. S27).

### Optimization of Single-Cell Structure

**Fig. 5:**
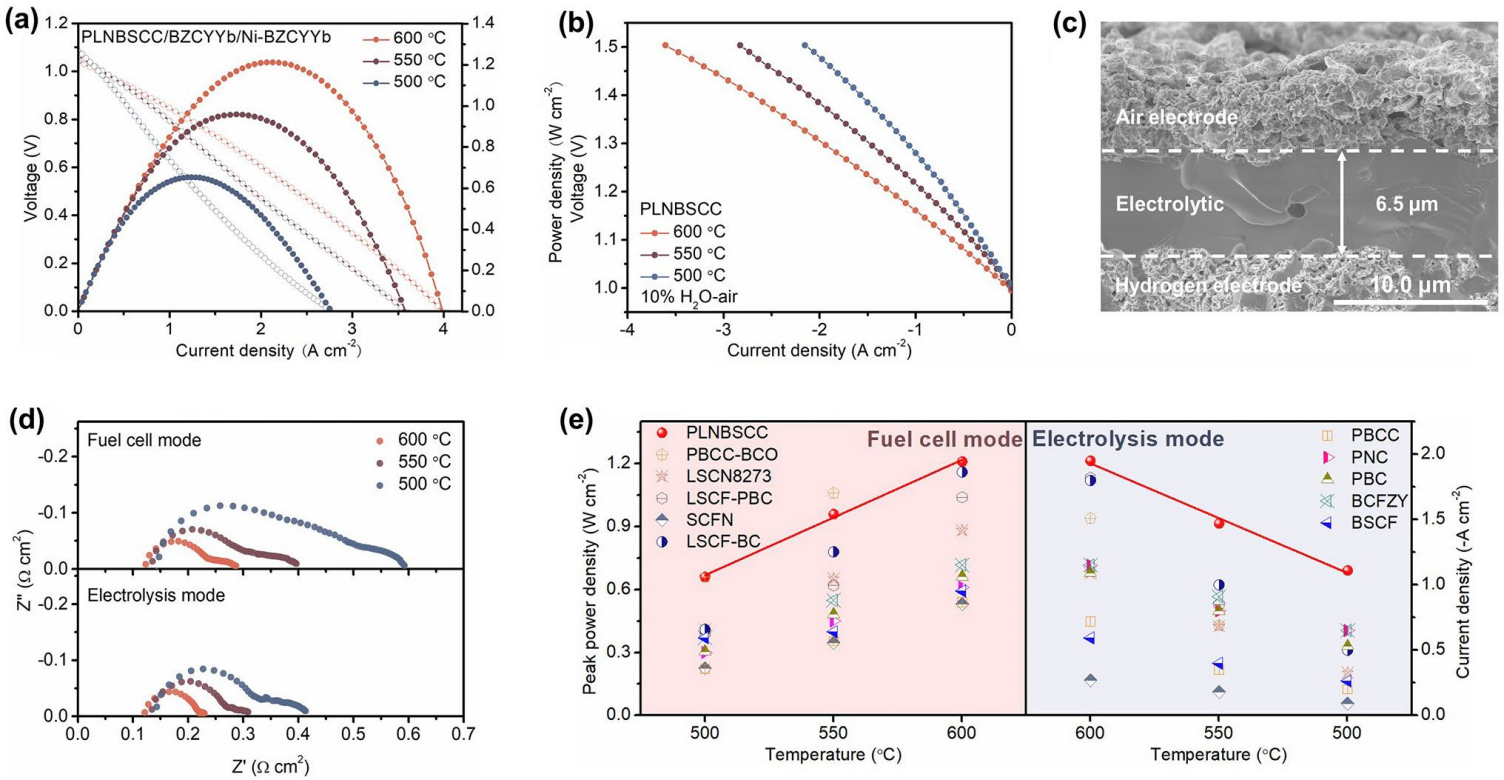
The single-cell structure was optimized by a spin-coating method to prepare thinner electrolyte layer and obtain excellent electrochemical performance. **a** *I*–*V* and power density curves during fuel cell operation and **b** *I*–*V* curves during electrolysis operation (10% H_2_O–air) were measured using an R-PCEC with high-entropy PLNBSCC air electrode at 500–600 °C. **c** Cross-sectional SEM image of single cell. **d** EIS curves of a single cell in fuel cell (open circuit voltage) and electrolysis modes (1.3 V) at 500–600 °C. **e** The peak power densities and current densities obtained by R-PCEC with PLNBSCC air electrode in fuel cell and electrolysis modes compared with some reported air electrode materials

The electrochemical performance of R-PCECs is limited mainly due to the large *R*_p_ caused by the slow kinetic rates of ORR and OER at the air electrodes combined with the ohmic resistance (*R*_o_) dominated by the electrolyte. The designed high-entropy PLNBSCC air electrode has been demonstrated to exhibit excellent ORR and OER activity. Here, a thinner electrolyte layer was prepared by spin coating, which further improved the electrochemical performance, optimized the cell structure, and reduced *R*_o_ [70, 71]. Figure 5a depicts the *I*–*V* and power density curves of the single cell using PLNBSCC air electrode and spin-coated electrolyte layer, the peak power densities of the single cell in fuel cell mode were 1.21, 0.96 and 0.66 W cm^−2^ at 600, 550 and 500 °C, respectively. In addition, the current densities measured in the electrolysis mode were − 1.95, − 1.47 and − 1.11 A cm^−2^ (1.3 V), respectively (Fig. 5b). Compared with the single cell prepared by the dry pressing method, the electrochemical performance is significantly improved. For example, the electrolytic performance is improved by 27.5% at 600 °C. Figure 5c exhibits the cross-sectional SEM morphology of the single cell prepared by the spin-coating method after testing [72]. The dense electrolyte layer with a thickness of about 6.5 μm was in close contact with the air and fuel electrodes [73, 74]. Meanwhile, the EIS curves of R-PCECs for fuel cell operation (open circuit voltage condition) and electrolysis operation (1.3 V) are shown in Fig. 5d. The *R*_o_ values were 0.12, 0.13 and 0.14 Ω cm^2^ for fuel cell mode at 600, 550 and 500 °C, respectively, while the *R*_p_ values were 0.17, 0.27 and 0.46 Ω cm^2^. The extremely low *R*_p_ and *R*_o_ are attributed to the excellent electrochemical activity of the high-entropy PLNBSCC and the thinner electrolyte layer, respectively. Therefore, compared with the advanced air electrodes reported so far, such as PrBa_0.8_Ca_0.2_Co_2_O_5+*δ*_-BaCoO_3−*δ*_ (PBCC-BCO), La_0.8_Sr_0.2_Co_0.7_Ni_0.3_O_3−*δ*_ (LSCN8273), (La_0.6_Sr_0.4_)_0.95_Co_0.2_Fe_0.8_O_3−*δ*_-Pr_1−*x*_Ba_*x*_CoO_3−*δ*_ (LSCF-PBC), Sr_0.9_Ce_0.1_Fe_0.8_Ni_0.2_O_3−*δ*_ (SCFN), La_0.6_Sr_0.4_Co_0.2_Fe_0.8_O_3−*δ*_-BaCoO_3−*δ*_ (LSCF-BC), (PrBa_0.8_Ca_0.2_)_0.95_Co_2_O_6−*δ*_ (PBCC) and PNC electrodes [4, 19, 39, 67, 75–77]. PLNBSCC air electrode exhibits one of the highest electrochemical performances in R-PCECs (Fig. 5e and Table S4).

## Conclusion

In summary, a cubic high-entropy perovskite oxide PLNBSCC was designed and successfully synthesized by performing A-site doping based on the classical PBC prototype by a facile sol–gel method, which demonstrates significantly enhanced ORR and OER activities as air electrodes for R-PCECs. The enhanced electrochemical performance is attributed to the promotion of the overall triple conductivity and hydration by various trivalent rare earth elements and divalent alkaline earth metal elements at the A-site. Not only that, the significant improvement in the structural stability of the PLNBSCC air electrode driven by the increase in effective entropy enables the single cell to perform well in long-term stability, thermal cycling tolerance, high water pressure tolerance and bifunctional cycling tests. Meanwhile, spin coating was used to prepare a thinner electrolyte layer to optimize the cell structure, enabling the high-entropy PLNBSCC oxide as a bifunctional R-PCEC air electrode to exhibit excellent electrochemical performance in the intermediate temperature range. This work not only demonstrates an outstandingly active and durable R-PCEC air electrode, but also opens a new boulevard for developing advanced high-entropy structured R-PCEC electrodes.

## Supplementary Information

Below is the link to the electronic supplementary material.

Supplementary file1 (PDF 3169 KB)
