# Supplementary material for: High-Entropy Perovskite Oxide: A New Opportunity for Developing Highly Active and Durable Air Electrode for Reversible Protonic Ceramic Electrochemical Cells
Source: Nanomicro Lett. 2022 Nov 9;14:217. doi: 10.1007/s40820-022-00967-6 (PMC9646682; doi:10.1007/s40820-022-00967-6)
Supplement: Supplementary file 1 — Supplementary file1 (PDF 3169 KB) [file 40820_2022_967_MOESM1_ESM.pdf]

Supporting Information for

High Entropy Perovskite Oxide: A New Opportunity for Developing Highly Active and Durable Air Electrodes for Reversible Protonic Ceramic Electrochemical Cells

Zuoqing Liu[1], Zhengjie Tang[1], Yufei Song[2], Guangming Yang[1,*], Wanru Qian[1], Meiting Yang[1], Yinlong Zhu[3,*], Ran Ran[1], Wei Wang[1], Wei Zhou[1], and Zongping Shao[1,4,*]

[1] State Key Laboratory of Materials-Oriented Chemical Engineering, College of Chemical Engineering, Nanjing Tech University, Nanjing, 211816, P. R. China

[2] Department of Mechanical and Aerospace Engineering, The Hong Kong University of Science and Technology, Clear Water Bay, Hong Kong, 999077, P. R. China

[3] Institute for Frontier Science, Nanjing University of Aeronautics and Astronautics, Nanjing 210016, P. R. China

[4] WA School of Mines: Minerals, Energy and Chemical Engineering (WASM-MECE), Curtin University, Perth, WA 6845, Australia

*Corresponding authors. E-mail: ygm89525@njtech.edu.cn (Guangming Yang); zhuyl1989@nuaa.edu.cn (Yinlong Zhu); shaozp@njtech.edu.cn (Zongping Shao)

Supplementary Figures

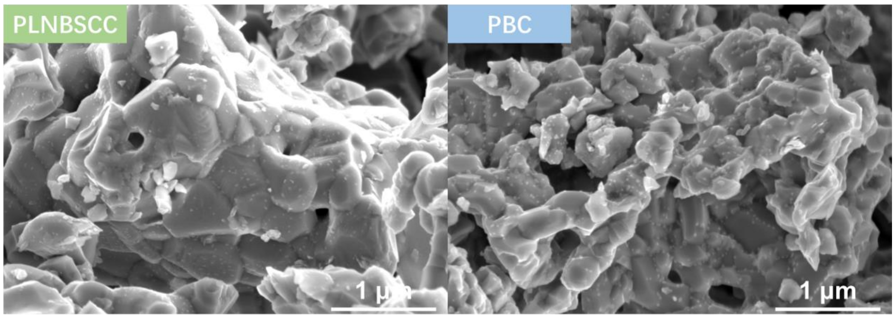

Fig. S1 SEM images of PLNBSCC and PBC powders

The morphologies of the synthesized PLNBSCC and PBC powders were basically the same, while the particle size of PLNBSCC was larger and has better sinterability, indicating that the increase of the entropy of PLNBSCC is beneficial to improve the stability of the structure and reduce the sintering temperature.

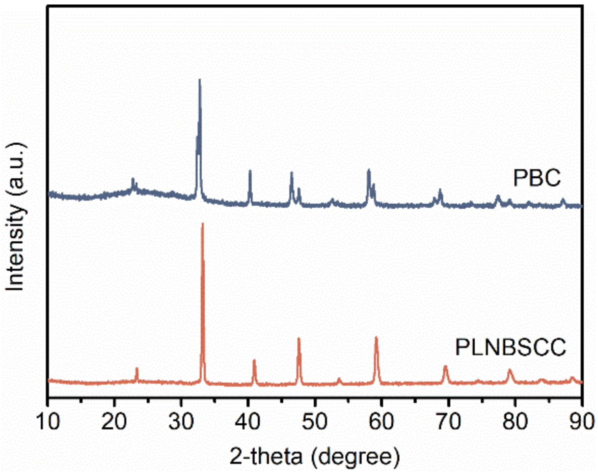

Fig. S2 XRD patterns of the PLNBSCC and PBC air electrodes

Both PLNBSCC and PBC powders were calcined at 1000 °C for 5 h. The XRD pattern can clearly find that the PLNBSCC powder has a cubic perovskite structure, while the PBC oxide exhibits typical double perovskite structure, and without the appearance of any additional peaks.

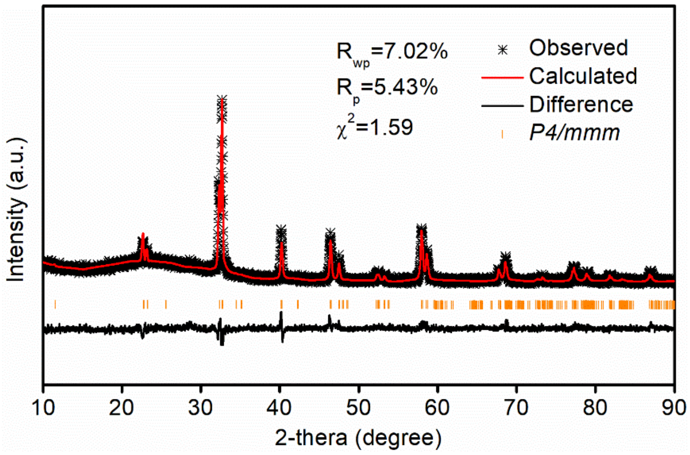

Fig. S3 Refined XRD profile of PBC powder

From the Rietveld refinement of the corresponding XRD pattern, the lattice parameters of a = b = 3.9103(6) Å, c = 7.6557(6) Å (R_p = 5.43%, R_{wp} = 7.02%, χ^2 = 1.59) were achieved for PBC [S1, S2].

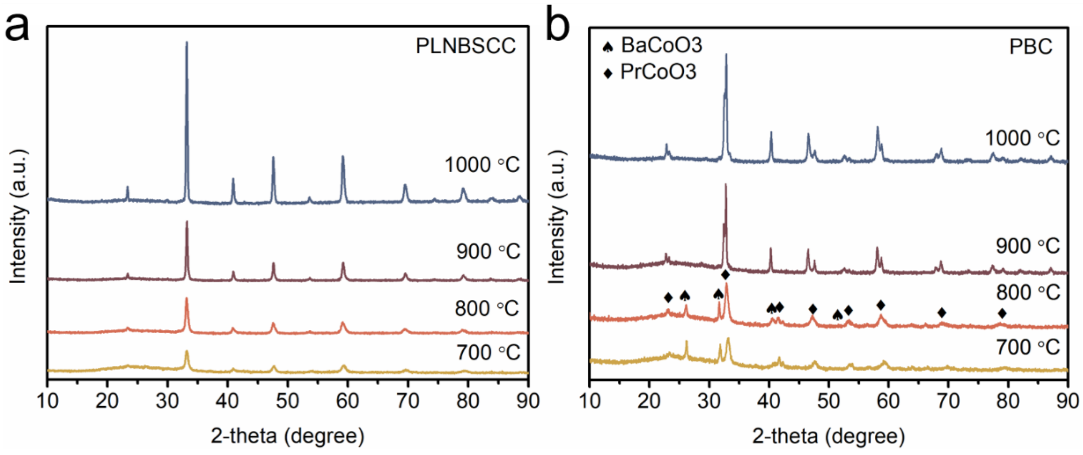

Fig. S4 XRD patterns of **a** PLNBSCC and **b** PBC powders calcined at 700, 800, 900 and 1000 °C for 5 h, respectively

In order to verify that the increase of entropy can effectively enhance the stability of the structure. Here, the PLNBSCC and PBC precursors were calcined at 700, 800, 900 and 1000 °C for 5 h, respectively. The XRD patterns reveal that the PLNBSCC oxides exhibit a cubic perovskite structure at all calcination temperatures. It is worth noting that the XRD patterns of PBC oxides calcined at 700 and 800 °C showed obvious characteristic peaks of $BaCoO_3$ and $PrCoO_3$, while PBC exhibited a double perovskite structure at 900 and 1000 °C. The lower phase formation temperature of PLNBSCC shows that the increase of configuration entropy is conducive to the stability of the structure.

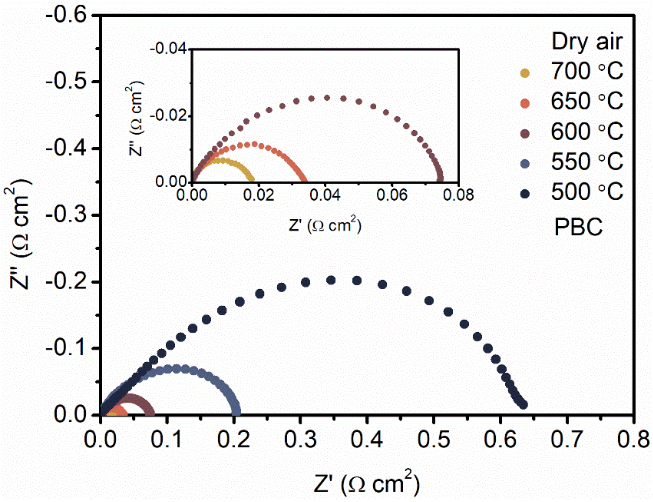

Fig. S5 EIS curves of GDC electrolyte-supported symmetrical cell with PBC measured at 500, 550, 600, 650 and 700 °C

The oxygen reduction reaction (ORR) activity of PBC electrodes at intermediate temperatures was first assessed by symmetric cells. As shown in Fig. S5, area-specific resistance (ASR) values of 0.019, 0.034, 0.075, 0.208 and 0.640 Ω cm^2 at 700, 650, 600, 550 and 500 °C, respectively, in air.

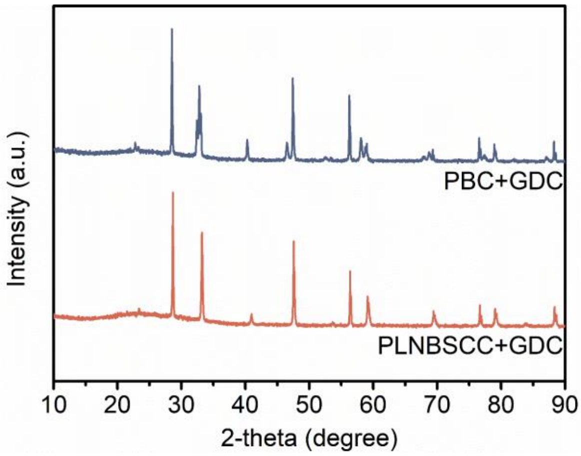

Fig. S6 XRD patterns of PLNBSCC and PBC powders mixed with GDC at a weight ratio of 1:1 and calcined at 1000 °C for 100 h

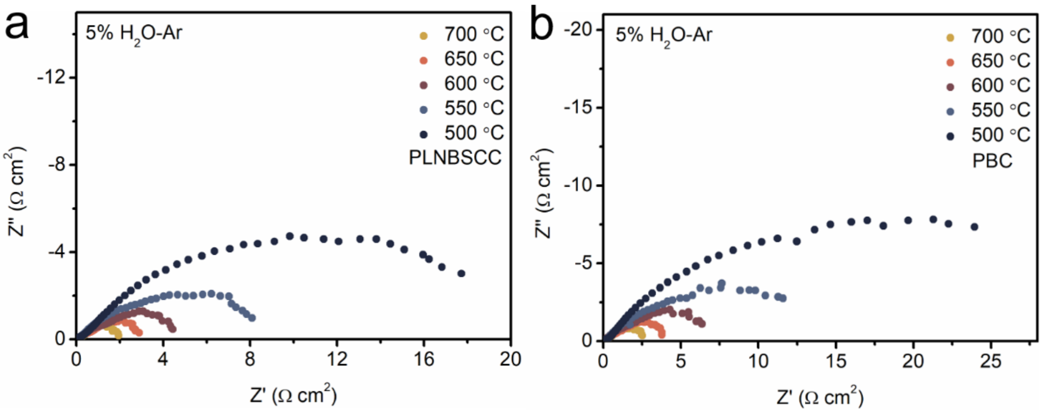

Fig. S7 EIS curves of BZCYYb electrolyte-supported symmetrical cells with **a** PLNBSCC, and **b** PBC measured in 5% H_2O-Ar at 500-700 °C

To compare the hydration reactions and proton conduction capabilities of PLNBSCC and PBC air electrodes, the BZCYYb electrolyte-supported symmetric cells with PLNBSCC and PBC

air electrodes were tested under 5% H_2O-Ar conditions. The symmetric cell with PLNBSCC air electrode shows very low ASRs of 2.0, 3.0, 4.7, 9.0 and 21 Ω cm^2 at 700, 650, 600, 550 and 500 °C, respectively. Compared with the cell using a bare PBC electrode with 2.5, 3.8, 7.5, 16.0 and 36.0 Ω cm^2 at 700, 650, 600, 550 and 500 °C, respectively, the ASR of the PLNBSCC air electrode decreased by about 37%, at 600 °C. This shows that the hydration capacity of PLNBSCC air electrode is significantly improved compared with that of PBC.

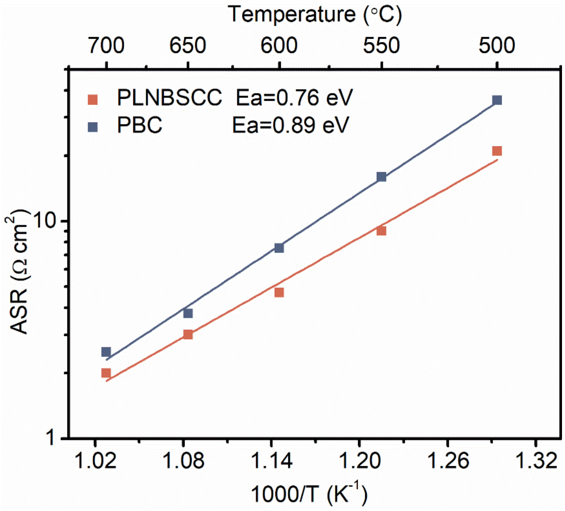

Fig. S8 Arrhenius plots of the ASRs of PLNBSCC and PBC air electrodes with BZCYYb-based symmetrical cells in 5% H_2O-Ar at 700-500 °C

According to the Arrhenius plot of ASR under 5% H_2O-Ar, PLNBSCC oxide exhibits lower ASR and activation energy than PBC oxide, indicating that the hydration reaction of high-entropy PLNBSCC electrode has lower dependence on temperature, and also shows excellent hydration ability at low temperatures.

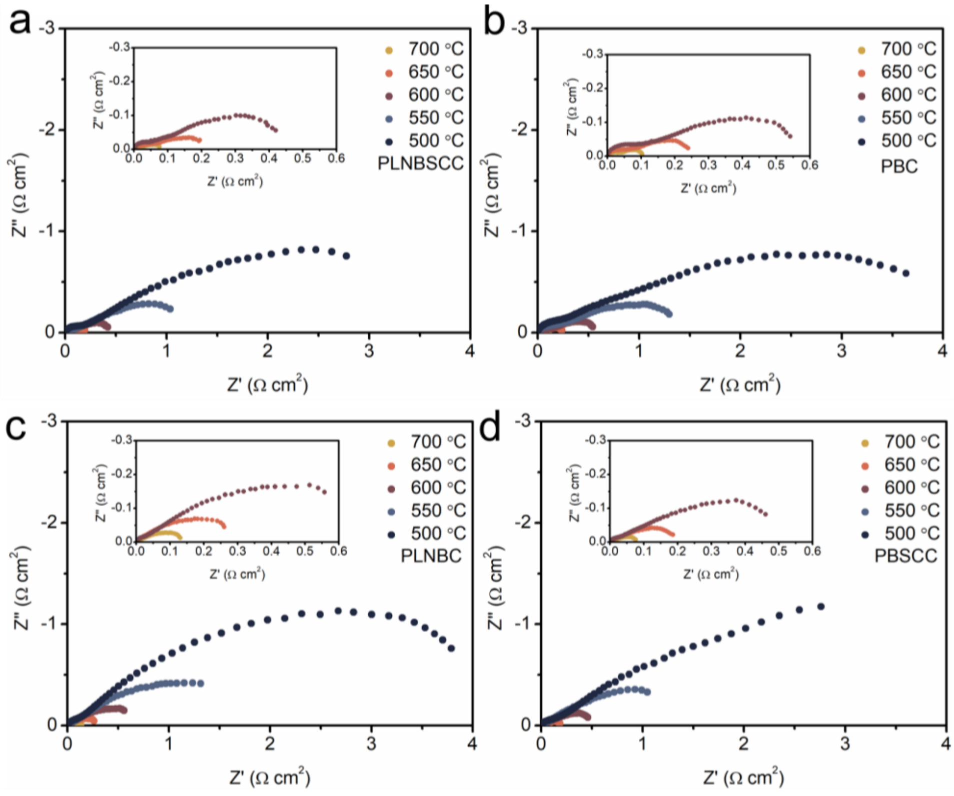

Fig. S9 The EIS curves of **a** PLNBSCC, **b** PBC, **c** PLNBC and **d** PBSCC air electrodes measured in dry air at 500-700 ℃

The ORR performance of PLNBSCC as R-PCEC air electrode is demonstrated from the ASR, which was achieved via EIS in a symmetrical cell under dry air. As shown in Fig. S9a, the obtained R_p of symmetric cells with PLNBSCC electrodes are 0.07, 0.21, 0.45, 1.23 and 3.5 Ω cm^2 at 700, 650, 600, 550 and 500 °C, respectively. The ASR is significantly lower than that of the PBC air electrode. For example, the ASR of PLNBSCC at 600 °C is 20% lower than that of PBC (0.56 Ω cm^2). It is shown that its ORR activity on proton-conducting symmetric cell is also better than that of PBC, which is the same as that obtained for GDC electrolyte-supported symmetric cells. While the EIS of PLNBC and PBSCC air electrodes are also obtained in Fig. S9c-d.

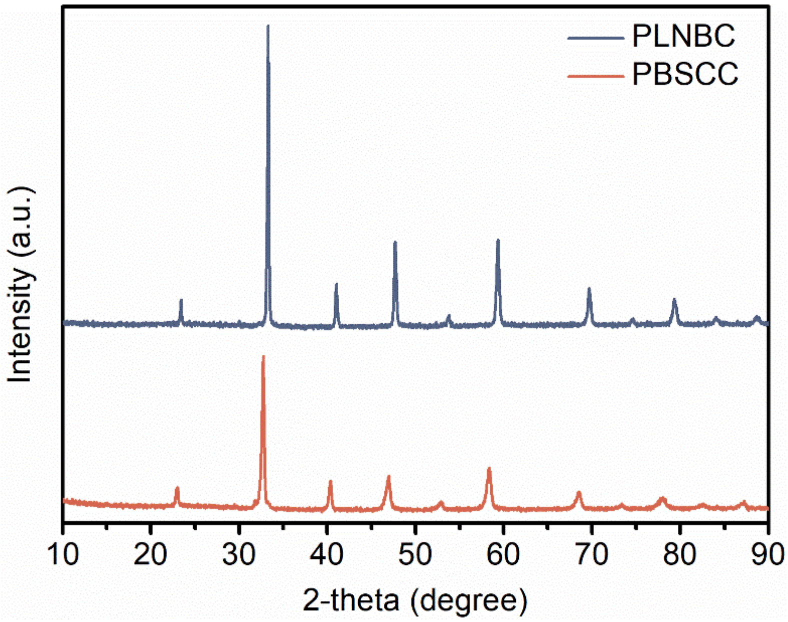

Fig. S10 XRD patterns of the PLNBC and PBSCC air electrodes. Both PLNBC and PBSCC powders were calcined at 1000 °C for 5 h

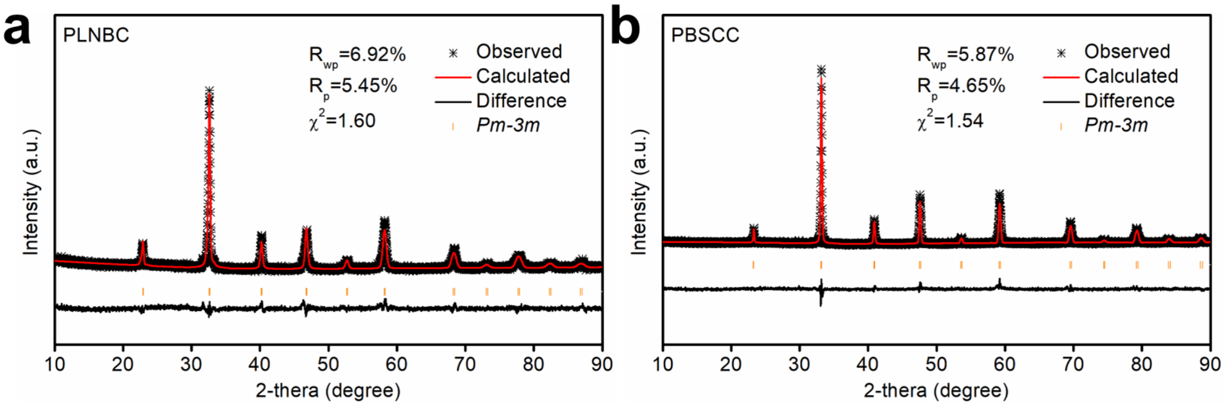

Fig. S11 Refined XRD profiles of **a** PLNBC and **b** PBSCC oxides

As shown in Fig. S11a, XRD refinement data further revealed that PLNBC is a space group of *Pm-3m* with lattice parameters of a = b = c = 3.8832(1) Å (R_p = 5.45%, R_{wp} = 6.92%, χ^2 = 1.60). Meanwhile, the PBSCC oxide also was shown a typical cubic perovskite structure, space group *Pm-3m*, with a = b = c = 3.8216(8) Å after Rietveld refinement (Fig. S11b). The reliability of the Rietveld refinement was confirmed by R_p = 4.65%, R_{wp} = 5.87%, χ^2 = 1.54, indicating a reliable fitting.

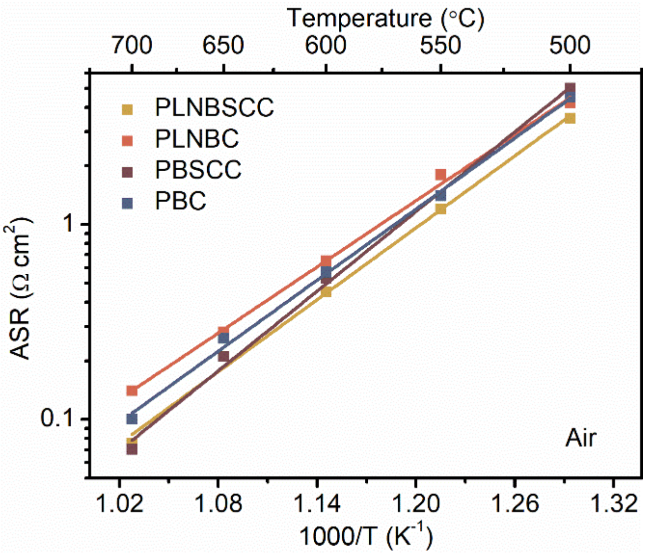

Fig. S12 Arrhenius plots of the ASRs of PLNBSCC, PLNBC, PBSCC and PBC air electrodes with BZCYYb-based symmetrical cells in air at 700-500 °C

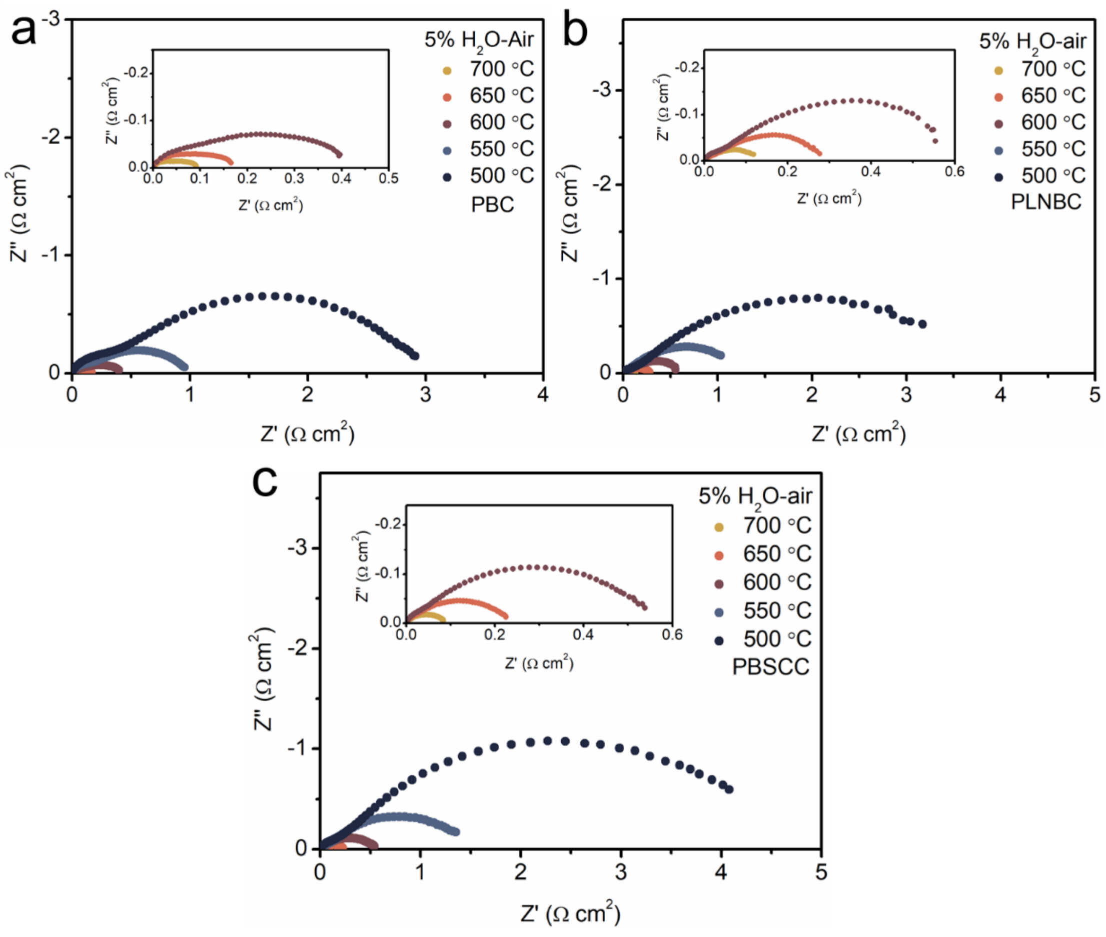

Fig. S13 EIS curves of **a** PBC, **b** PLNBC and **c** PBSCC air electrodes measured in 5% H_2O-air at 500-700 °C

The EIS curves of PBC, PLNBC and PBSCC air electrodes were acquired at 5% H_2O-air at 500-700 °C, as shown in Fig. S12a, the R_p of PBC electrode were 0.09, 0.17, 0.42, 1.00 and 3.00 Ω cm^2 at 700, 650, 600, 550 and 500 °C, respectively. Comparing the R_p values can clearly analyze that PBC has better electrochemical activity compared to PLNBC and PBSCC air electrodes.

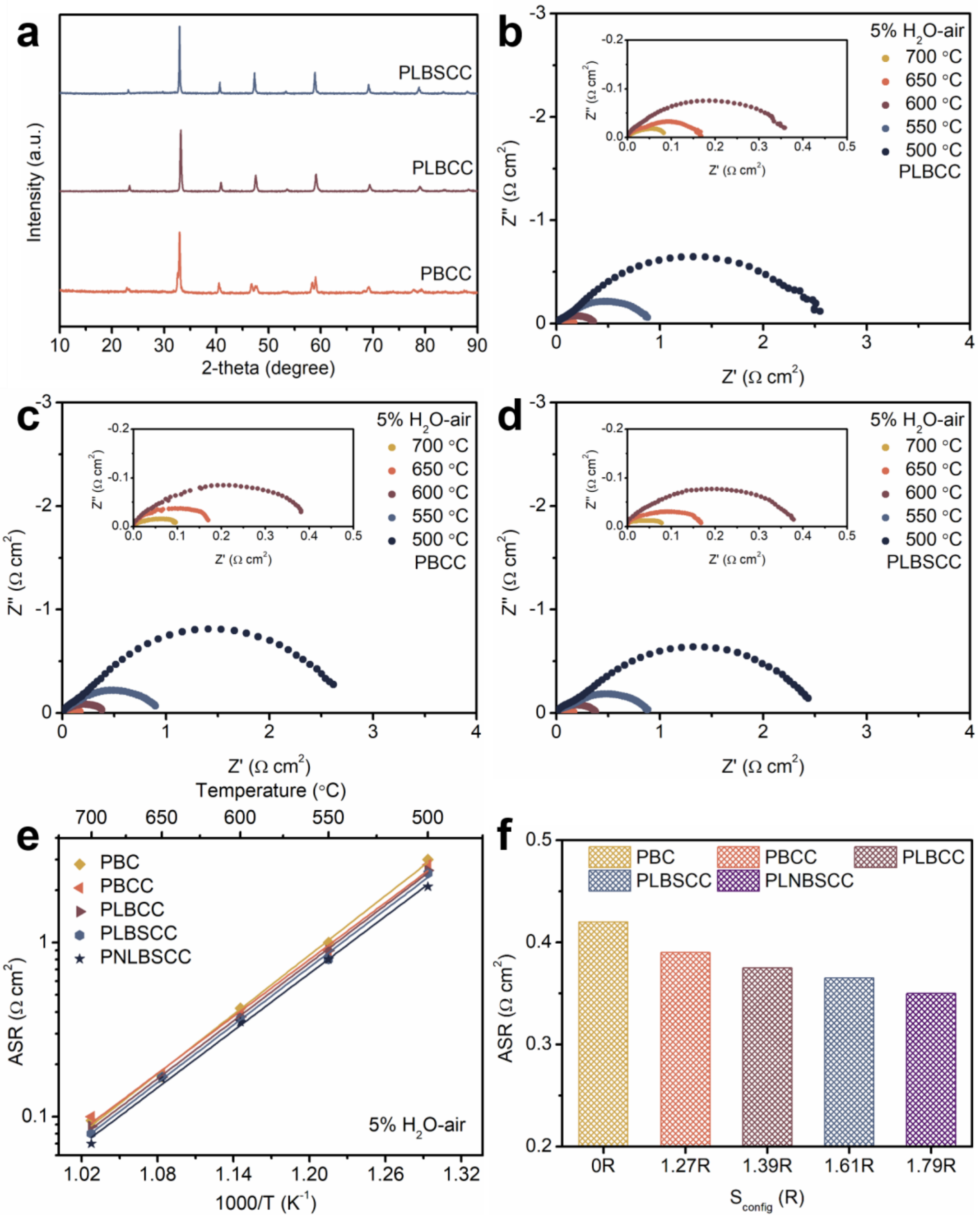

Fig. S14 a XRD patterns of the synthesized PBCC, PLBCC and PLBSCC oxides at room temperature. Typical EIS spectra or the symmetrical cells with the **b** PBCC, **c** PLBCC and **d** PLBSCC electrodes tested in 5% H_2O-air at 700-500 °C. **e** Arrhenius plots of the ASR in 5% H_2O-air. **f** Configuration entropy and measured ASRs of different air electrodes under 5%H_2O-air condition at 600 ℃

The EIS of $(Pr_{1/3}Ba_{1/3}Ca_{1/3})_2Co_2O_{5+\delta}$ (PBCC), $Pr_{1/4}La_{1/4}Ba_{1/4}Ca_{1/4}CoO_{3-\delta}$ (PLBCC) and $Pr_{1/5}La_{1/5}Ba_{1/5}Sr_{1/5}Ca_{1/5}CoO_{3-\delta}$ (PLBSCC) electrodes, and combined with PBC and PLNBSCC electrodes to discuss (Fig. S14b, c and d). Based on the XRD patterns, PBCC exhibits double perovskite structure, while PLBCC and PLBSCC exhibit cubic perovskite structure (Fig. S14a). It is revealed that the transition of the crystal structure tends to a more stable state with the increase of entropy. At the same time, with the introduction of Ca, La, Sr and Nd in the A-site of PBC oxide one by one, and ensuring the same proportion of each element in the A-site, the ASR measured by PBC, PBCC, PLBCC, PLBSCC and PLNBSCC electrodes gradually

decreased under humid air, indicating the improvement of electrochemical activity (Fig. S14e). The results show that the configurational entropy of the electrode is gradually increased, which is accompanied by the stabilization of the structure and the improvement of the activity (Fig. S14f).

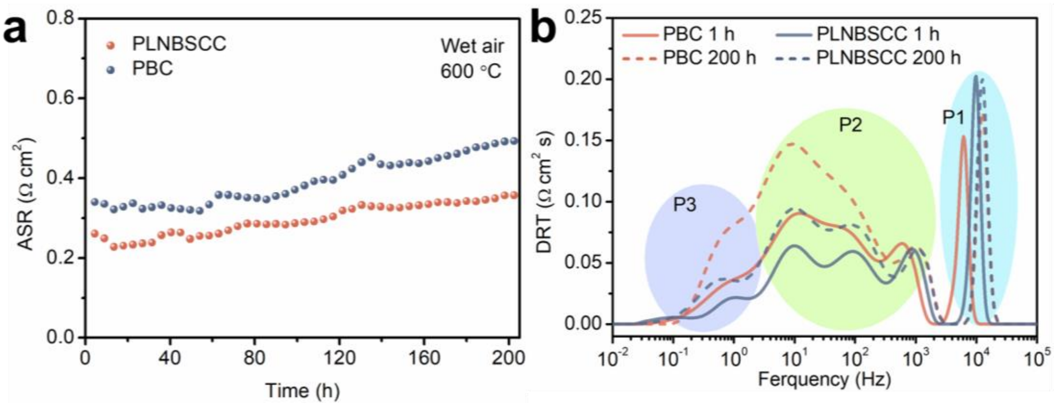

Fig. S15 a Time dependence of the electrode polarization resistance (Rp) of the symmetrical cells with the PLNBSCC and PBC electrodes measured in 5% H_2O-air at 600 °C under open-circuit conditions. **b** DRT analysis of EIS of the PBC and PLNBSCC air electrodes at the beginning and end of the stability test

The first and last EIS during the stability test were analyzed by the DRT tool to gain a clearer understanding of the key factors in the stability decay of different air electrodes. In DRT plots, three distinct frequency segments, denoted as high frequency (>2000 Hz, P1), intermediate frequency (10-2000 Hz, P2), and low frequency (< 10 Hz, P3), correspond to ion transfer from the electrolyte to the electrode at the TPB, surface exchange or ion transfer (H^+ and O^{2-}) at the electrode bulk, and gas diffusion (H_2O(g) and O_2), respectively. The DRT analysis results of the PLNBSCC electrodes showed slight changes in the P2 region before and after the stability test. On the other hand, the deconvoluted peak areas of the P3 and P2 regions of the PBC air electrode increased significantly, which may be related to the poor structural stability of the PBC, which produced more barium hydroxide on the surface in humid air, resulting in slow surface mass transfer (P3) , ion exchange and bulk diffusion (P2) in humid atmospheres.

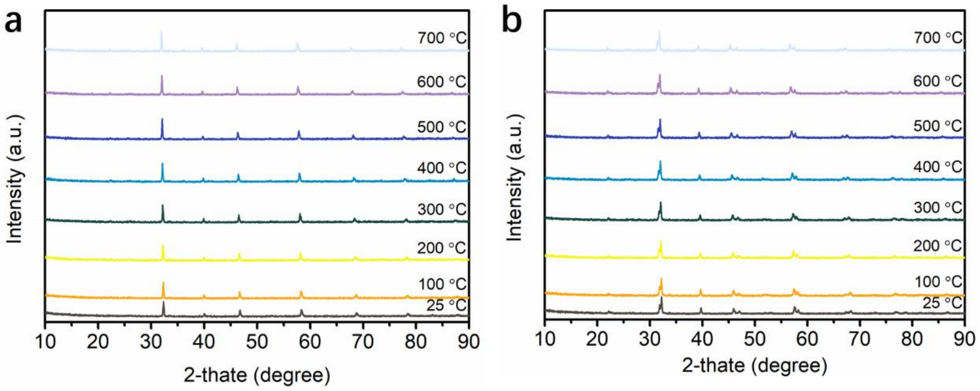

Fig. S16 High-temperature *in-situ* XRD patterns of **a** PLNBSCC and **b** PBC powders

Through high-temperature *in-situ* XRD, it can be clearly found that PLNBSCC and PBC powders still maintain good crystal structure in the tested temperature range, and as the temperature increases, the characteristic peaks shift to low angles due to lattice expansion.

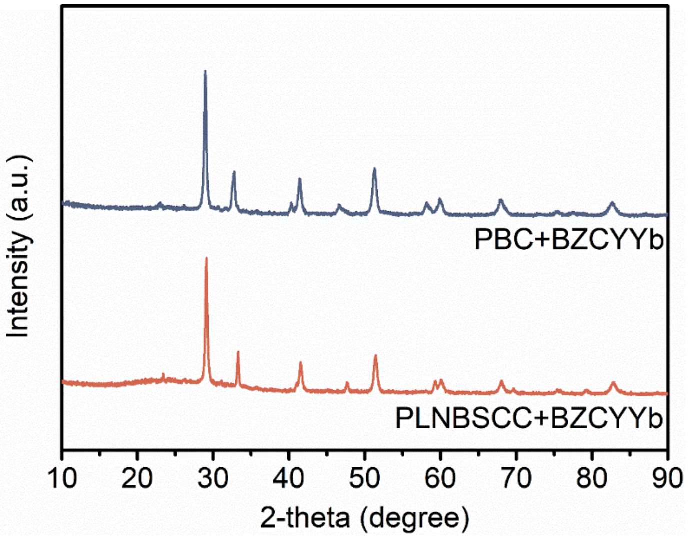

Fig. S17 XRD patterns of PLNBSCC and PBC powders mixed with BZCYYb at a mess ratio of 1:1 and calcined at 1000 °C for 100 h

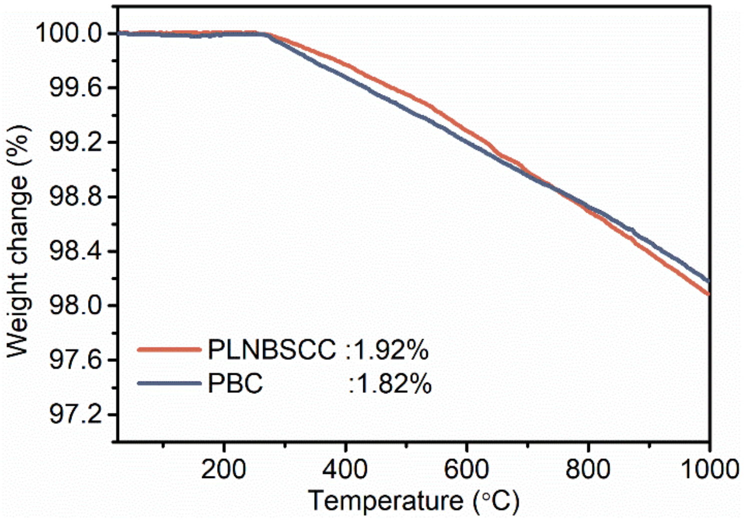

Fig. S18 TG curves of PLNBSCC and PBC powders in the temperature range from room temperature to 1000 °C in dry air

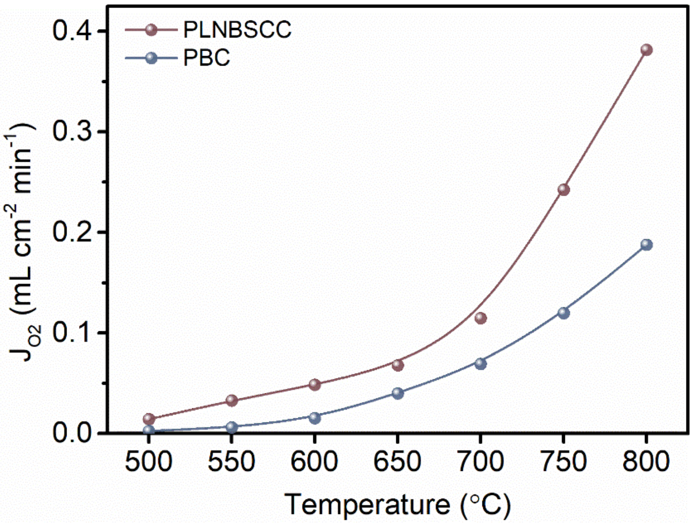

Fig. S19 Oxygen penetration rate of PLNBSCC and PBC oxides from 500 to 800 °C

Oxygen permeation measurements were performed by exposing PLNBSCC and PBC discs (0.7 mm thick) on one side to still air and the other side to a sweep of high-purity Ar (sweep rate of

60 sccm). As shown in Fig. S17, the oxygen permeability gradually increased with the increase of temperature, and the oxygen permeability of PLNBSCC was much higher than that of PBC in the tested temperature range, indicating that it has higher oxygen ionic conductivity. However, oxygen ionic conductivity can be calculated by Eq. S1.

$$\sigma_{O^{2-}} = J_{O2}\frac{4F^2L}{RT} \Big/ \ln\frac{P_{O2,supp.}}{P_{O2,perm.}} \tag{S1}$$

Where J_{O2} is O_2 permeation flux (mol cm^{-2} s^{-1}), F is the Faraday constant (96485.3326 C mol^{-1}), L is the membrane thickness, R is the ideal gas constant (8.314 J K^{-1} mol^{-1}), T is the temperature, $P_{O2,\ supp.}$ is O_2 partial pressure at the feed side, and $P_{O2,\ perm.}$ is O_2 partial pressure at the permeate side.

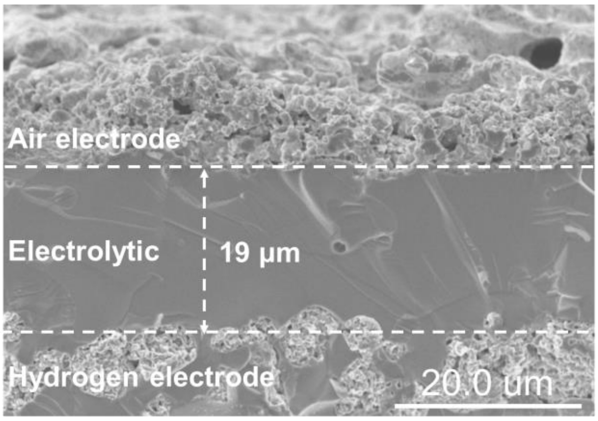

Fig. S20 Cross-section SEM image of single cell with PLNBSCC electrode

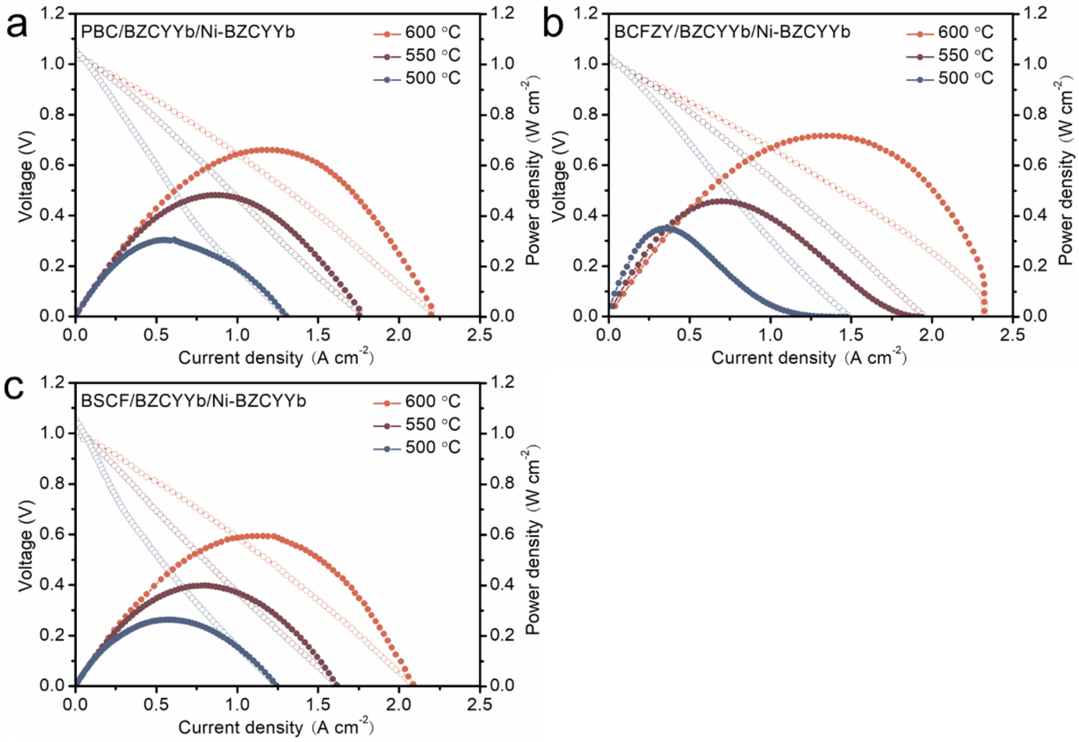

Fig. S21 *I-V* and power density curves of the single cells with **a** PBC, **b** BCFZY and **c** BSCF as air electrodes at 500-600 °C

Evaluating the electrochemical performance of PBC air electrodes in fuel cell mode, a Ni-BZCYYb anode-supported cell with a BZCYYb electrolyte and PBC electrode was fabricated. The *I-V* and power density curves of the PBC air electrode were also acquired as shown in Fig.

S19a, with peak power densities (PPDs) of 0.66, 0.48 and 0.31 W cm^{-2} at 600, 550 and 500 °C, respectively. In order to better compare the electrochemical performance of the PLNBSCC air electrode with the reported electrode materials, the *I-V* and power density curves of the popular BCFZY and BSCF air electrodes were also measured. The PPDs of BCFZY was higher than that of PBC electrode by 0.72, 0.55 and 0.37 W cm^{-2} at 600, 550 and 500 °C, respectively. However, the air electrode BSCF, which is a classical mixed conduction of oxygen ions and electrons, exhibits poor electrochemical performance.

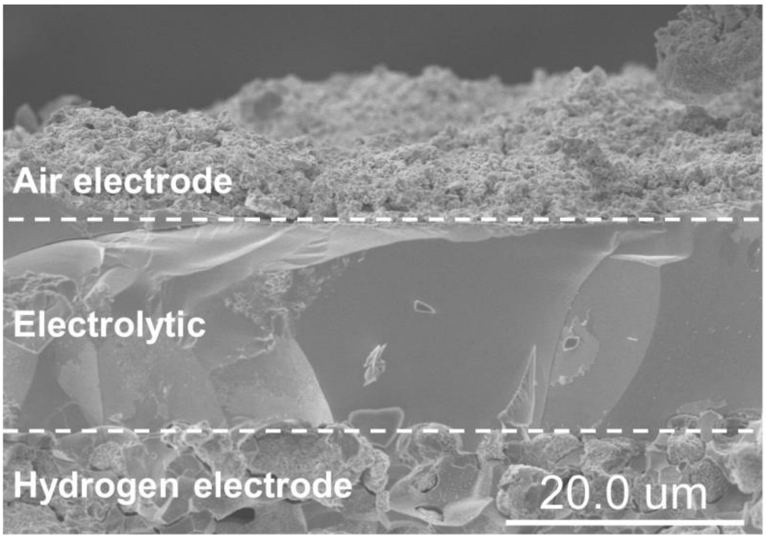

Fig. S22 Cross-section SEM image of single cell after stability test

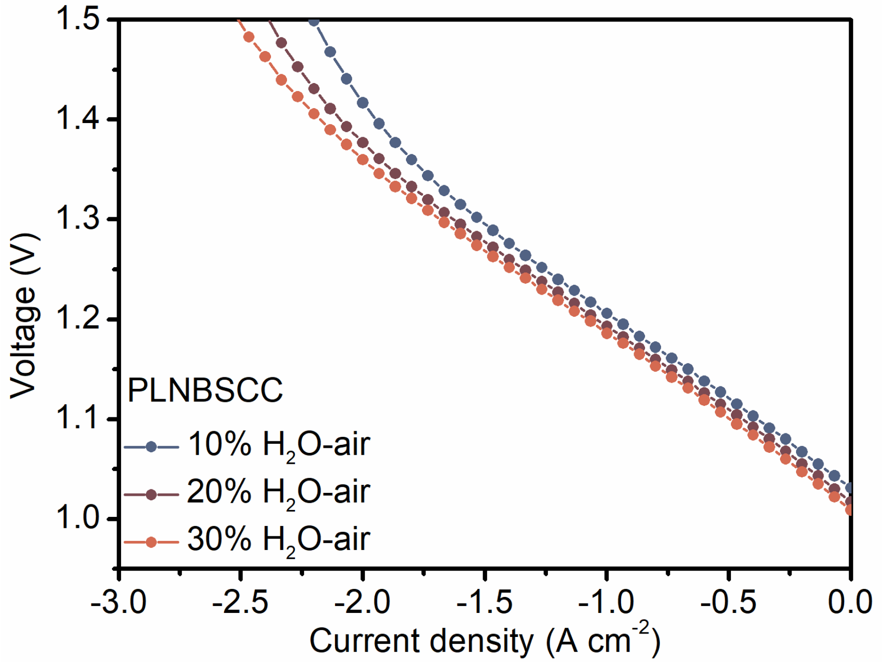

Fig. S23 Typical *I-V* curves of single cell with PLNBSCC electrode tested in electrolysis mode under different water pressure conditions (10%, 20% and 30% H_2O-air) at 600 °C

Figure S23 shows typical *I-V* curves of a single cell with a PLNBSCC air electrode in electrolysis mode at 600 °C. At 10% 20% and 30% H_2O-air, the current densities of electrolysis corresponding to a voltage of 1.3 V were -1.53, -1.67 and -1.73A cm^{-2}, respectively, indicating that the performance of electrolysis mode increases slightly with increasing water pressure.

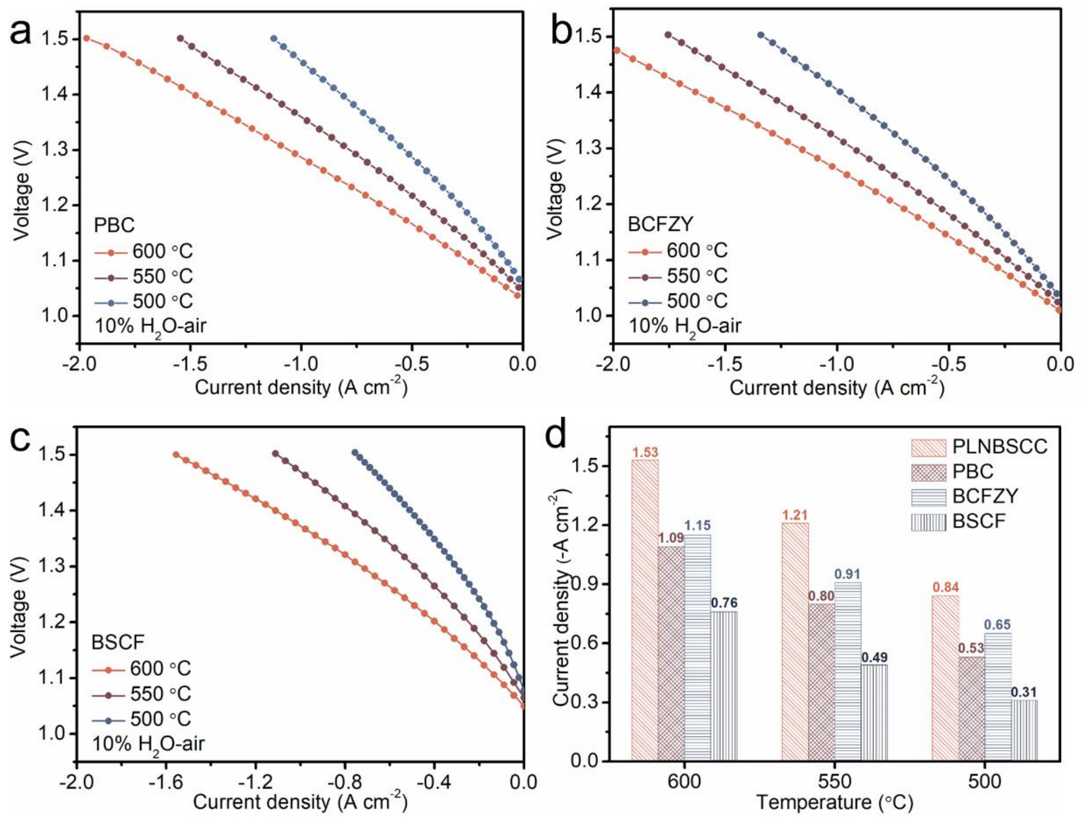

Fig. S24 *I-V* curves of single cells with **a** PBC, **b** BCFZY and **c** BSCF electrodes measured in electrolysis mode at 500-600 °C. **d** Current densities of PLNBSCC air electrode in electrolysis mode compared to PBC, BCFZY and BSCF air electrodes

PBC, BCFZY and BSCF as air electrodes for R-PCECs were also tested in electrolysis mode. Fig. S24 shows the *I-V* curves of the single cells at 500-600 °C when the fuel electrode was exposed to dry hydrogen and the air electrode supplied with humidified air (10% H_2O-air). The current densities of PBC electrode were -1.09, -0.80 and -0.53 A cm^{-2} at 600, 550 and 500 °C, respectively, and the cell voltage was 1.3 V. Compared with PBC, BCFZY and BSCF, PLNBSCC has excellent electrolytic performance. For example, at 600 °C, the current density of PLNBSCC is -1.53 A cm^{-2}, compared with PBC, BCFZY and BSCF air electrodes (-1.09, -1.15 and -0.76 A cm^{-2}), which are improved by 40%, 33% and 101%, respectively. Such excellent electrolytic performance of the high-entropy PLNBSCC air electrode is attributed to the excellent electronic conductivity, hydration ability and the OER activity.

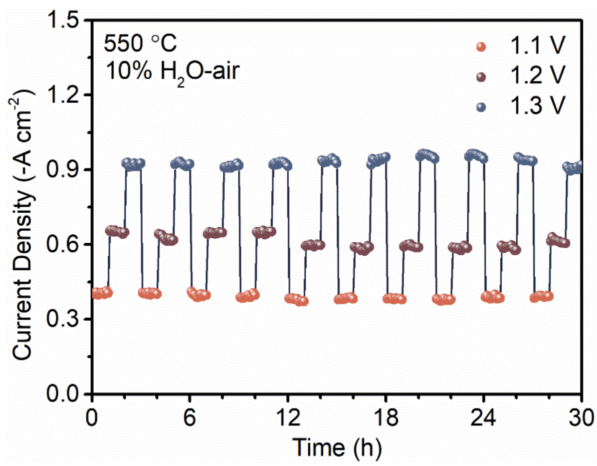

Fig. S25 Cycling test of the single cell with PLNBSCC electrode at different voltages in electrolysis mode at 550 °C

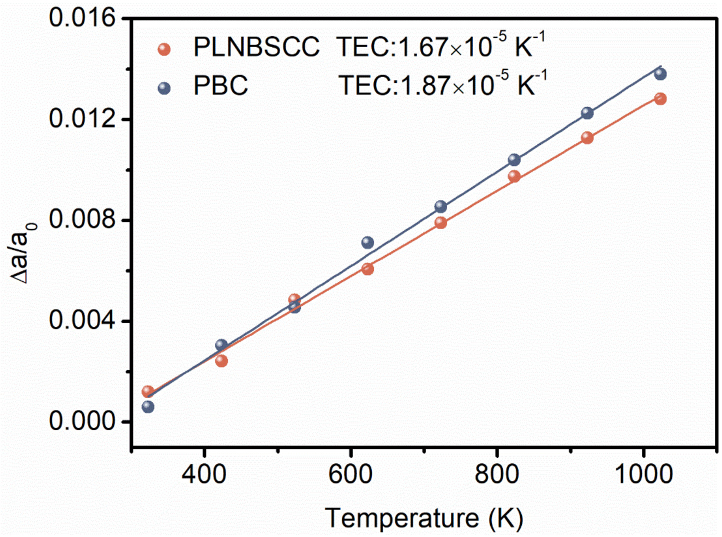

Fig. S26 TEC profiles of PLNBSCC and PBC oxides determined from high-temperature XRD data

According to the high-temperature XRD patterns of PLNBSCC and PBC powders, and the interplanar spacing under different temperature conditions is calculated by the Bragg equation ($2d\sin\theta = n\lambda$). d is the interplanar spacing, θ is the angle, x-ray wavelength n=1 (first-order diffraction), and λ is the wavelength. The thermal expansion coefficient of PLNBSCC is (1.67×10^{-5} K^{-1}) lower than that of PBC (1.87×10^{-5} K^{-1}), indicating that the high-entropy PLNBSCC has better thermal matching with the electrolyte.

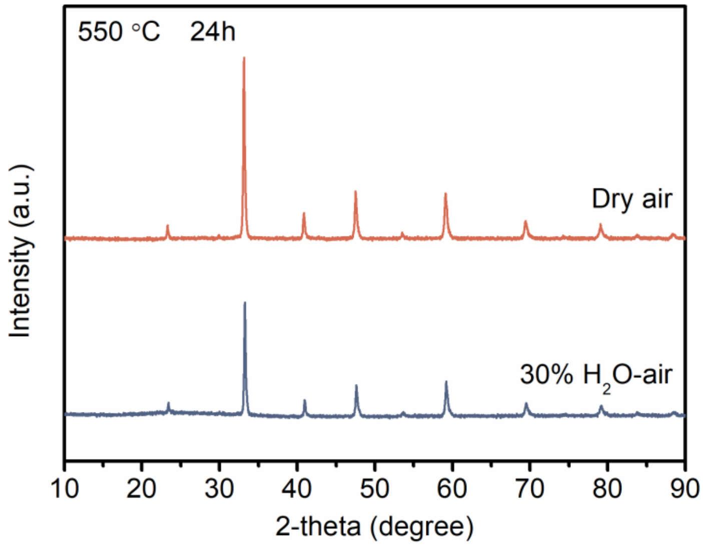

Fig. S27 XRD patterns of the high-entropy perovskite oxide PLNBSCC calcined at 550 °C for 24 h in dry air and 30% H_2O-air atmosphere

Table S1 The XRD refinements information of PLNBSCC and PBC samples

Sample	Crystal parameters		Phase proportions (wt%)	Refinement parameters
PLNBSCC	Cubic (*Pm-3m*)	a=3.8301 (3)<br>b=3.8301 (3)<br>c=3.8301 (3)	100	Rp=3.51<br>Rwp=4.55<br>x^2=1.79
PBC	Layered (*P4/mmm*)	a=3.9103 (6)<br>b=3.9103 (6)<br>c=7.6557 (6)	100	Rp=5.43<br>Rwp=7.02<br>x^2=1.59

Table S2 R_p values of symmetrical cells with PLNBSCC, PLNBC, PBSCC and PBC electrodes in the temperature range of 500-700 °C under 5% H_2O-air

	R_p (Ω cm^2)				
	700 °C	650 °C	600 °C	550 °C	500 °C
PLNBSCC	0.07	0.16	0.35	0.81	2.09
PLNBC	0.13	0.29	0.56	1.25	3.80
PBSCC	0.09	0.22	0.57	1.60	4.75
PBC	0.10	0.17	0.42	1.00	3.00

Table S3 Comparing the ASRs obtained by air electrodes with different configurational entropies on symmetric cells under the conditions of 5% H_2O-air at 500-700 °C

Air electrode	S_{config}	Electrochemical performance (ASR: Ω cm^2)				
		700 °C	650 °C	600 °C	550 °C	500 °C
PBC	0R	0.95	0.18	0.42	1.01	3.02
PBCC	1.27R	0.10	0.18	0.39	0.92	2.81
PLBCC	1.39R	0.09	0.17	0.38	0.91	2.65
PLBSCC	1.61R	0.08	0.17	0.37	0.82	2.53
PLNBSCC	1.79R	0.07	0.16	0.35	0.79	2.10

Table S4 Comparison of electrochemical performance of R-PCECs with different air electrodes in fuel cell mode and electrolysis mode

Year	Air electrode	Electrolyte	Electrochemical performance						Refs.
			Fuel cell mode (W cm^{-2})			Electrolysis mode (-A cm^{-2})			
			600 °C	550 °C	500 °C	600 °C	550 °C	500 °C	
2022	PLNBSCC	BZCYYb	1.21	0.96	0.66	1.95	1.47	1.11	This work
	PBC	BZCYYb	0.662	0.482	0.305	1.09	0.8	0.53	
	BCFZY	BZCYYb	0.717	0.549	0.37	1.15	0.91	0.65	
	BSCF	BZCYYb	0.593	0.399	0.37	0.593	0.399	0.263	
2022	LSCF-PBC	BZCYYb	1.04	0.62	0.39	1.82	0.85	-	[S3]
2021	PBCC-BCO	BZCYYb	-	1.06	0.66	1.51	0.69	-	[S4]
2021	LSCN8273	BZCYYb	0.882	0.652	0.409	1.09	0.684	0.327	[S5]
2021	SCFN	BZCYYb	0.531	0.347	0.225	0.269	0.179	0.091	[S6]
2021	LSCF-BC	BZCYYb	1.16	0.78	0.41	1.8	1	0.5	[S7]
2020	PBCC	BZCYYb	0.54	0.354	0.222	0.72	0.35	0.2	[S8]
2020	PNC	BZCYYb	0.611	0.45	0.3	1.15	0.8	0.65	[S9]

LSCF-PBC: $(La_{0.6}Sr_{0.4})_{0.95}Co_{0.2}Fe_{0.8}O_{3-\delta}$-$Pr_{1-x}Ba_xCoO_{3-\delta}$, PBCC-BCO: $PrBa_{0.8}Ca_{0.2}Co_2O_{5+\delta}$-$BaCoO_{3-\delta}$, LSCN8273: $La_{0.8}Sr_{0.2}Co_{0.7}Ni_{0.3}O_{3-\delta}$, SCFN: $Sr_{0.9}Ce_{0.1}Fe_{0.8}Ni_{0.2}O_{3-\delta}$, LSCF-BC: $La_{0.6}Sr_{0.4}Co_{0.2}Fe_{0.8}O_{3-\delta}$-$BaCoO_{3-\delta}$, PBCC: $(PrBa_{0.8}Ca_{0.2})_{0.95}Co_2O_{6-\delta}$, PNC: $PrNi_{0.5}Co_{0.5}O_{3-\delta}$.

Supplementary References

[S1] B.V. Politov, A.Y. Suntsov, I.A. Leonidov, M.V. Patrakeev, V.L. Kozhevnikov, High-temperature defect thermodynamics of nickel substituted double-perovskite cobaltite $PrBaCo_{2-x}Ni_xO_{6-\delta}$ (x = 0.2). J. Alloys Compd. **727**, 778-784 (2017). https://doi.org/10.1016/j.jallcom.2017.08.063

[S2] C. Lim, A. Jun, H. Jo, K.M. Ok, J. Shin et al., Influence of Ca-doping in layered perovskite $PrBaCo_2O_{5+\delta}$ on the phase transition and cathodic performance of a solid oxide fuel cell. J. Mater. Chem. A **4**(17), 6479-6486 (2016). https://doi.org/10.1039/C6TA00746E

[S3] Y. Niu, Y. Zhou, W. Zhang, Y. Zhang, C. Evans et al., Highly active and durable air electrodes for reversible protonic ceramic electrochemical cells enabled by an efficient bifunctional catalyst. Adv. Energy Mater. **12**(12), 2103783 (2022). https://doi.org/10.1002/aenm.202103783

[S4] Y. Zhou, E. Liu, Y. Chen, Y. Liu, L. Zhang et al., An active and robust air electrode for reversible protonic ceramic electrochemical cells. ACS Energy Lett. **6**(4), 1511-1520 (2021). https://doi.org/10.1021/acsenergylett.1c00432

[S5] N. Wang, H. Toriumi, Y. Sato, C. Tang, T. Nakamura et al., $La_{0.8}Sr_{0.2}Co_{1-x}Ni_xO_{3-\delta}$ as the efficient triple conductor air electrode for protonic ceramic cells. ACS Appl. Energy Mater. **4**(1), 554-563 (2020). https://doi.org/10.1021/acsaem.0c02447

[S6] Y. Song, J. Liu, Y. Wang, D. Guan, A. Seong et al., Nanocomposites: a new opportunity for developing highly active and durable bifunctional air electrodes for reversible protonic ceramic cells. Adv. Energy Mater. **11**(36), 2101899 (2021). https://doi.org/10.1002/aenm.202101899

[S7] Y. Zhou, W. Zhang, N. Kane, Z. Luo, K. Pei et al., An efficient bifunctional air electrode for reversible protonic ceramic electrochemical cells. Adv. Funct. Mater. **31**(40), 2105386 (2021). https://doi.org/10.1002/adfm.202105386

[S8] W. Tang, H. Ding, W. Bian, W. Wu, W. Li et al., Understanding of A-site deficiency in layered perovskites: promotion of dual reaction kinetics for water oxidation and oxygen reduction in protonic ceramic electrochemical cells. J. Mater. Chem. A **8**(29), 14600-14608 (2020). https://doi.org/10.1039/D0TA05137C

[S9] H. Ding, W. Wu, C. Jiang, Y. Ding, W. Bian et al., Self-sustainable protonic ceramic electrochemical cells using a triple conducting electrode for hydrogen and power production. Nat. Commun. **11**, 1970 (2020). https://doi.org/10.1038/s41467-020-15677-z
